# An EEG Analysis of Honorification in Japanese: Human Hierarchical Relationships Coded in Language

**DOI:** 10.3389/fpsyg.2021.549839

**Published:** 2021-03-08

**Authors:** Shingo Tokimoto, Yayoi Miyaoka, Naoko Tokimoto

**Affiliations:** ^1^Department of English Language Studies, Mejiro University, Tokyo, Japan; ^2^Faculty of Liberal Arts, Hiroshima University of Economics, Hiroshima, Japan; ^3^Department of Policy Management, Shobi University, Saitama, Japan

**Keywords:** Japanese honorification, social cognition, human relationship, event- related potential, event-related spectral perturbation, N400, dipole fitting, independent component clustering

## Abstract

This study examines the neural substrate of the understanding of human relationships in verbal communication with Japanese honorific sentences as experimental materials. We manipulated two types of Japanese verbs specifically used to represent respect for others, i.e., exalted and humble verbs, which represent respect for the person in the subject and the person in the object, respectively. We visually presented appropriate and anomalous sentences containing the two types of verbs and analyzed the electroencephalogram elicited by the verbs. We observed significant parietal negativity at a latency of approximately 400 ms for anomalous verbs compared with appropriate verbs. This parietal negativity could be a manifestation of the pragmatic process used to integrate the linguistic forms with the human relationships represented in the sentences. The topographies of these event-related potentials (ERPs) corresponded well with those of ERPs for two second-person pronouns in Chinese (plain *ni* and respectful *nin*). This correspondence suggests that the pragmatic integration process in honorific expressions is cross-linguistically common in part. Furthermore, we assessed the source localization by means of independent component (IC) analysis and dipole fitting and observed a significant difference in ERP between the honorific and control sentences in the IC cluster centered in the precentral gyrus and in the cluster centered in the medial part of the occipital lobe, which corresponded well with the functional magnetic resonance imaging findings for Japanese honorification. We also found several significant differences in the time-frequency analyses for the medial occipital cluster. These significant differences in the medial occipital cluster suggested that the circuit of the theory of mind was involved in the processing of Japanese honorification. Our results suggest that pragmatic and syntactic processing are performed in parallel because the person to be respected must fulfill the grammatical function appropriate for the honorific verb.

## 1. Introduction: Honorification as Social Cognition

Many neuroscientists and cognitive scientists have begun to pay substantial attention to the mental and neural mechanisms of human social behavior. Verbal communication is one such social behavior, and politeness is a linguistic phenomenon that is a direct manifestation of human relationships. While politeness is universal in human communication, the linguistic forms used to express politeness vary depending on the relationship between the speaker and the addressee(s), that is, their gender, the relative social power, and the intimacy between them (Brown and Levinson, 1978). Furthermore, the effects of these aspects of human relationships can differ in strength among languages and cultures; therefore, politeness is a suitable phenomenon for examining the universal and language-specific properties of human social behavior.

The ways of expressing politeness vary among languages and cultures, and politeness is often communicated by indirect expressions. Several examples in English presented in (1) can be used to communicate the intention of the speaker to ask the addressee to open a window. The intention of the speaker is directly expressed in (1-a), whereas (1-b-f) are examples in which the intention is communicated indirectly.

(1)    a.    Open the window.
         b.    Could you open the window?
         c.    I wonder if you could open the window.
         d.    I was wondering if you could open the window.
         e.    Would you mind if I asked you to open the window?
         f.    It's too hot, isn't it?

The number of indirect expressions to communicate an intention is infinite in principle; therefore, we can easily find many indirect expressions other than (1) to communicate the intention that is directly expressed in (1-a). Furthermore, we can find no one-to-one correspondence between the literal meaning of a linguistic form and the speaker's intention. Moreover, we can recognize the different degrees of politeness in (1), and the degree can vary depending on the relationship between the speakers and the context. While the variability in indirect expression to express politeness is one manifestation of the productivity of human language, the variability in politeness is a major reason why an experimental study of politeness can be difficult to manipulate.

The Japanese language has many words specific to expressing respect for others; moreover, the linguistic forms of honorification can be categorized differently according to the social relationship between the speaker and the addressee(s) to be respected. The honorification used in Japanese is thus appropriate for an experimental study on politeness. In this study, we examined the neural substrate of honorification as a social cognition with Japanese honorific expression as the experimental material. In the following, we will briefly introduce the characteristics of the Japanese language and its honorific expressions.

Japanese is agglutinative and head-final. The grammatical function of a noun phrase is indicated by a case-particle with the correspondence to the verb. The word order in a clause is free as long as the main verb is placed at the end of a sentence. Japanese has three types of linguistic form used to show respect, namely, polite, exalted, and humble expressions. The polite form is used to represent the resupect to a non-argument addresses, as shown in (2-b). In (2), *ki-mashi-ta* (came) in (2-b) expresses the respect extended to the non-argument addressee, while the propositional meanings of the two sentences are the same.


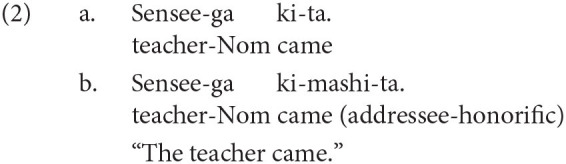


This type of honorification corresponds to allocutivity in Basque. Some Basque examples are reproduced in (3) from Antonov (2015).


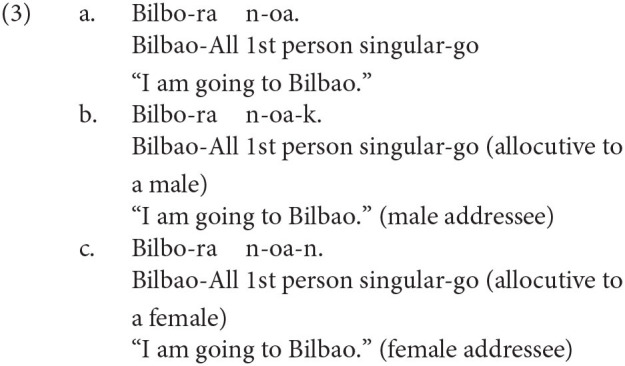


The linguistic forms of addressee-honorifics can also be found in Korean. The polite form for a non-argument addressee is not the focus of the current study.

The other two honorific forms, namely, the exalted and the humble forms are used to represent the respect for the person in the subject and that for the person in the object as in (4-a) and (5-a), respectively. The mental and neural processing of these two forms is the main focus of the current study. Some examples of exalted expressions are presented in (4). The exalted verb *osshai-mashita* (said) in (4) expresses respect for the person in the subject [*sensee* (teacher) in (4-a)], who is senior to the speaker. The function of an exalted verb can thus be understood as subject honorification. Therefore, when the speaker [*watashi* (I)] is placed in the subject of an exalted verb, the sentence is judged anomalous, as in (4-b) (Top, Gen, Acc, and Dat in (4) are abbreviations for topic, genitive, accusative, and dative, respectively).


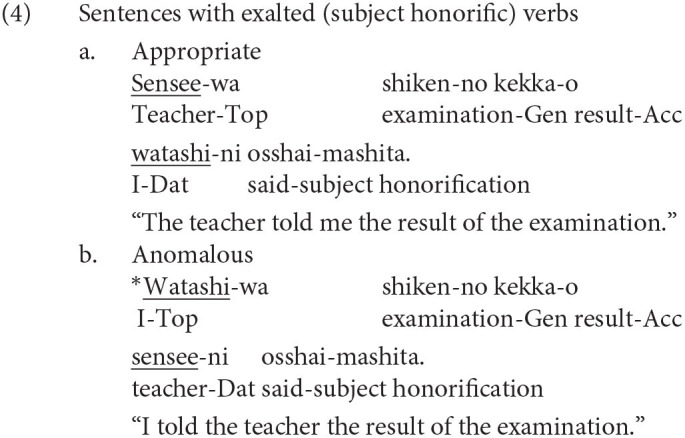


On the other hand, in (5), the humble verb *mooshiage-mashita* (said) represents respect for the person in the object [*sensee* in (5-a)], who should be senior to the speaker and the person in the subject. The function of a humble verb can thus be understood as object honorification. The speaker and the person in the subject are identical in (5-a), and the subject can be replaced with another third person to ensure that the sentence is appropriate as long as we can naturally assume that the status of the person in the object is higher than the one in the subject (for example, my younger brother in the subject and his teacher in the object). The example (5-b) is anomalous because the teacher (*sensee*), who is superior to the speaker, is in the subject, whereas the speaker (*watashi*) is placed in the object to be respected by the verb.


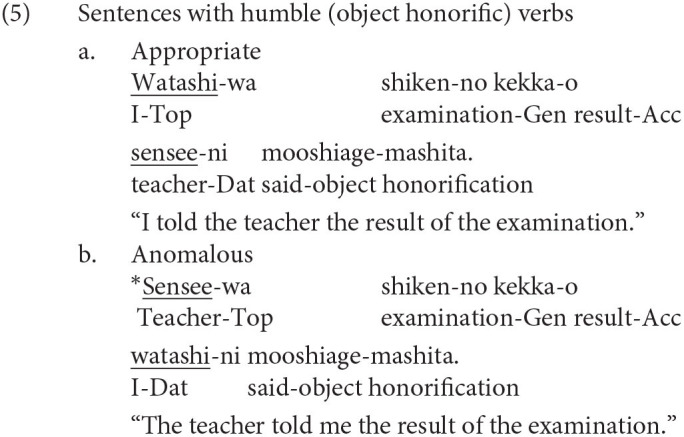


We can manipulate the appropriateness of an exalted (subject honorific) and a humble (object honorific) sentence by replacing an exalted verb with a humble verb and vice versa. We can thus expect that the contrast of neural activity between appropriate and anomalous sentences will be a manifestation of the processes of human social relationships. We will refer to exalted and humble sentences as subject-honorific and object-honorific sentences in the following discussion.

Jiang et al. (2013) is among the instructive studies used for predicting the results of our experiment. Many languages have two second-person singular pronouns, that is, a plain and informal form and a respectful and formal form. The distinction of *tu/vous* in French and that of *du/Sie* in German are two examples. Mandarin Chinese also has two second-person pronouns: *ni/ni-de* (you/your) is the plain form that presupposes an equal relationship between a speaker and the addressee or a relationship in which a speaker is superior to the addressee, and *nin/nin-de* (you/your) is the respectful form to express respect for the addressee. Jiang et al. (2013) changed the relative social status between the speaker and the addressee in the context to manipulate the appropriateness of the use of *ni/ni-de* or *nin/nin-de*, as in (6) and (7), respectively (originally in Chinese). The plain *ni-de* or the respectful *nin-de* in the directly quoted speech was either consistent or inconsistent with the social status of the speaker and the addressee described in the preceding context. That is, (6-a) is appropriate (status consistent) because the person (a professor) who is superior to the student refers to the student with the plain form *ni-de*, whereas (6-b) is disrespectful (status inconsistent) because the student, who is inferior to the professor, refers to the professor with *ni-de*.

(6)      Plain form: *ni/ni-de*
           a.     Status consistent
                   Professor Ye to Student Lü said to, “your (ni-de) situation I have heard about.”
                   (Professor Ye said to Student Lü, “I have heard about your_*plain*_ situation.”)
           b.     Status inconsistent (disrespectful)
                   Student Lü to Professor Ye said to, “your (ni-de) situation I have heard about.”
                   (Student Lü said to Professor Ye, “I have heard about your_*plain*_ situation.”)

On the other hand, (7-a) is status consistent because the student refers to the professor with the respectful form *nin-de*, whereas (7-b) is overly respectful (status inconsistent) because the professor refers to the student with the respectful form *nin-de*.

(7)      Respectful form: *nin/nin-de*
           a.     Status consistent
                   Student Liu to Professor Li said to, “your (nin-de) article I have finished reading.”
                   (Student Liu said to Professor Li, “I have finished reading your_*respectful*_ article.”)
           b.     Status inconsistent (overly respectful)
                   Professor Li to Student Liu said to, “your (nin-de) article I have finished reading.”
                   (Professor Li said to Student Liu, “I have finished reading your_*respectful*_ article.”)

Jiang et al. (2013) conducted electroencephalogram (EEG) analysis for *ni-de* and *nin-de* to examine the neural activity of the integration process between the linguistic forms and the human social statuses described. The Chinese sentences were visually presented to the participants segment by segment with a stimulus onset asynchrony (SOA) of 800 ms and an interstimulus interval (ISI) of 400 ms. The participants were presented with a probe statement [e.g., “Student Liu has finished reading the article written by Professor Li.” for (7) ]at the end of each sentence and were required to determine whether the statement was consistent or inconsistent with the message of the sentence by pressing a button. Jiang et al. (2013) analyzed event-related potentials (ERPs) that were time-locked to the onset of second-person pronouns. The status-inconsistent disrespectful use of *ni-de* in (6-b) elicited broadly distributed negativity in the time window of 300–500 ms and late negativity mainly in the left anterior, central, and right occipital regions in the time window of 500–1,800 ms compared with the status-consistent condition. On the other hand, the status-inconsistent overly respectful use of *nin-de* in (7-b) elicited anterior negativity in the time window of 300–500 ms and late positivity mainly in the parietal region in the time window of 500–1,800 ms compared to the status-consistent use of the pronoun.

A negative ERP with a peak latency of approximately 400 ms (N400) is one of the most frequently discussed ERP components known to indicate semantic processing (Kutas and Hillyard, 1980). Pragmatic processing is relatively new in the discussion of linguistic ERP. However, several previous studies have demonstrated that inconsistency between the critical word in an utterance and world knowledge or the social identity of the speaker elicited an N400 on the critical word (Hagoort et al., 2004; Hald et al., 2007; Van Berkum et al., 2009; van den Brink et al., 2012). Several other studies have also reported an N400 when the target word in an utterance was inconsistent with the social identity of the speaker (age, gender, or social status), which could be inferred from the prosody of the utterance (Van Berkum et al., 2008) and the N400 for the processing of pragmatic information encoded in the morphosyntax (Hanuĺıková and Carreiras, 2015).

Jiang et al. (2013) assumed that a processing difficulty arose in their status-inconsistent conditions due to the inconsistency between the Chinese pronouns and the world knowledge on the human relationship. That is, the second-person pronoun *ni/nin* in the directly quoted speech referred to the addressee, and the integration process between the pronoun and its antecedent (the addressee) became difficult when the linguistic form for the addressee conflicted with the relative social status of the addressee (hence, the N400).

According to Jiang et al. (2013), positivity was observed for the overly respectful *nin* [as in (7-b)] around a latency of 500 ms and could be elicited later by an inferential process that derived indirect meaning from the literal meaning of *nin*. That is, the overuse of the respectful form by a speaker of higher status referring to an addressee of lower status could be interpreted as an expression of non-literal meaning, as was observed in Coulson and Van Petten (2007) for metaphor and in Regel et al. (2010) for irony. As for the negativity observed for the disrespectful *ni* [as in (6-b)] around a latency of 500 ms or later, Jiang et al. (2013) suggested that a second-pass process to reinterpret the initially constructed semantic representation could elicit late negativity; that is, the participants might perceive the *ni* as a mistake that was not intended by the speaker and try to reinterpret the *ni* as *nin*.

Our anomalous sentences with subject-honorific verbs correspond to the overly respectful use of *nin/nin-de* in Jiang et al. (2013) because the speaker is inappropriately honored by the verb. On the other hand, our anomalous sentences with object-honorific verbs correspond to the disrespectful use of *ni/ni-de* because the person who is socially superior to the speaker is dishonored, relative to the speaker, by the verb.

In Japanese linguistics, researchers have long discussed the coding of the regularity observed in the use of honorific expressions, as shown in the contrast between (a) and (b) of (4) and (5). Specifically, a theoretical question has been posed regarding whether the regularity emerges from the syntactic constraints or from the semantic interpretation of honorific words. The relevant linguistic phenomenon is grammatical agreement; that is, a subject and/or an object agree(s) with the verb in person, number, and gender in many languages. However, Japanese verbs do not agree with subjects or objects; the correspondences between a subject-honorific verb and its subject and between an object-honorific verb and its object (and subject) can be understood as an exceptional case of agreement between a verb and its argument(s) in Japanese. A negative ERP is often observed for agreement errors in English in the left anterior region, which has been referred to as left anterior negativity (LAN) (Kutas and Hillyard, 1983; Münte et al., 1997; Coulson et al., 1998; Molinaro et al., 2015). As one of the early studies, Coulson et al. (1998) visually presented the sentences in (8) word by word with an SOA of 500 ms and an ISI of 300 ms and observed the LAN for *mow* in (8-a) and *suns* in (8-b) in the time window of 300-500 ms.

(8)    a.    Every Monday he {^*^mow/mows} the lawn.
         b.    They {^*^suns/sun} themselves on the beach (Coulson et al., 1998).

If the correspondence between an honorific verb and its subject and object in Japanese is neurally the same as the agreement between a verb and its subject in English, we can expect the LAN to occur for honorific verbs in the anomalous sentences compared to appropriate sentences.

We should note here that a semantic anomaly can elicit a positive ERP with a peak latency of approximately 600 ms (P600) though P600 is often understood to be a manifestation of syntactic processing. We find no syntactic violation in (9-b) as long as we assume the syntactic constraints to be constraints on the combination of parts of speech. (9-b) is anomalous due to a violation of selectional restriction that the subject of *eat* should be animate while it is inanimate *eggs*. Kuperberg et al. (2003) visually presented (9) word by word with an SOA as 400 ms and an ISI as 100 ms and observed P600 at *eat* for (9-b) against (9-a).

(9)    a.    No violation
                For breakfast the boys would only eat toast and jam.
         b.    Thematic role animacy violation
                For breakfast the eggs would only eat toast and jam.

Kim and Osterhout (2005) also observed a P600 at *devouring* in (10-c) against *devoured* in (10-a) and *devouring* in (10-b), though we find no syntactic violation in (10-c).

(10)     a.     Passive control
                    The hearty meal was devoured by the kids.
            b.     Active control
                    The hungry boy was devouring the cookies.
            c.     Violated
                    The hearty meal was devouring the kids.

If pragmatic processing is required for the inconsistency between the linguistic form and the relative social relationship between the persons described in the sentences, as was claimed by Jiang et al. (2013), we can predict an N400 for subject-honorific and object-honorific verbs in the anomalous sentences in (4) and (5), respectively. In contrast, if the regularity found in subject-honorific and object-honorific expressions is mentally coded as grammatical rules, a LAN will be observed. Furthermore, if an anomaly in Japanese honorific sentences is comparable to the semantic/thematic anomaly in (9) and (10), a P600 will be observed^1^.

It will be proper here to note that a LAN/N400 is often followed by a P600 (Hoeks et al., 2004; van Herten et al., 2006; Van Petten and Luka, 2006; van de Meerendonk et al., 2008; Kim and Sikos, 2011). One of our concerns here is the possible relationship between two ERP components. van de Meerendonk et al. (2008) visually presented Dutch sentences in (11), in which they changed one word in each of the sentences to manipulate their plausibility. That is, the sentence in (11-a) was a plausible grammatical sentence, and (11-b,c) were made to be semantically anomalous by replacing one word *netvlies* (retina) underlined in (11-a) with *wenkbrauw* (eyebrow) in (11-b) and with *sticker* (sticker) in (11-c).

(11)     a.     Plausible
                   Het oog bestaande uit onder andere een pupil, iris, en netvlies is erg gevoelig.
                   (The eye consisting of, among other things, a pupil, iris, and retina is very sensitive.)
            b.    Mildly implausible
                   Het oog bestaande uit onder andere een pupil, iris, en wenkbrauw is erg gevoelig.
                   (The eye consisting of, among other things, a pupil, iris, and eyebrow is very sensitive.)
            c.    Deeply implausible
                   Het oog bestaande uit onder andere een pupil, iris, en sticker is erg gevoelig.
                   (The eye consisting of, among other things, a pupil, iris, and sticker is very sensitive.)

An N400 was observed for *wenkbrauw* (eyebrow) in (11-b), and an N400 and a P600 were observed for *sticker* (sticker) in (11-c) compared to *netvlies* (retina) in (11-a). Our main concern here is the claim by van de Meerendonk et al. (2008) that the P600 counteracted the N400. More recently, Kim et al. (2018) observed an N400 and a P600 for *devouring* in (12-b,c) compared to (12-a) by the word-by-word visual presentation of the sentences in (12) [‘Semantic attraction’ in (12-b) means that the sentence can be appropriately interpreted via reanalysis to replace *devouring* with *devoured*. On the other hand, (12-c) cannot be plausible by replacing *devouring* with *devoured*, even though we find no violation in the parts of speech series].

(12)     a.     Control
                    The hearty meal was devoured with gusto.
            b.     Semantic attraction
                    The hearty meal was devouring with gusto.
            c.     No semantic attraction
                    The dusty tabletops were devouring with gusto.

The effect sizes of the N400 and P600 in (12-b,c) were significantly negatively correlated with each other. Kim et al. (2018) suggested that the N400 and P600 effects were functionally linked in a trade-off relationship. On the other hand, Tokimoto et al. (2019) analyzed the ERP elicited by two types of syntactic phenomena in Japanese, namely, syntactic integration of discontinuous dependency and syntactic island violation, and observed biphasic ERPs, negativity and subsequent positivity. The effect sizes of negativity and positivity were significantly positively correlated. That is, a reader who showed greater negativity also showed greater positivity. We can thus assume that a negative correlation in magnitude between negativity and positivity is characteristic of a semantic anomaly, whereas a positive correlation between them is a manifestation of syntactic processes. Therefore, if we observe biphasic ERPs for our anomalous sentences, their correlation can be informative for the neural coding of the regularity in honorific expressions in Japanese.

We will now turn to the relevance of our experiment to the findings by functional magnetic resonance imaging (fMRI) on linguistic honorification. Momo et al. (2008) is one of the few fMRI studies examining the neural activity of Japanese honorification. Adult linguistic knowledge is often assumed to be homogeneous among speakers in standard linguistic theory. However, we often recognize individual differences in the degree of fluency in honorification, even among adult native speakers. Momo et al. (2008) examined the individual differences in the development of linguistic knowledge in adults with Japanese honorific sentences as their experimental sentences. Some of the experimental sentences of Momo et al. (2008) are reproduced in (13) and (14). The examples in (13) are for the honorification task, and those in (14) are for the spelling judgment task (Nom is an abbreviation for nominative).


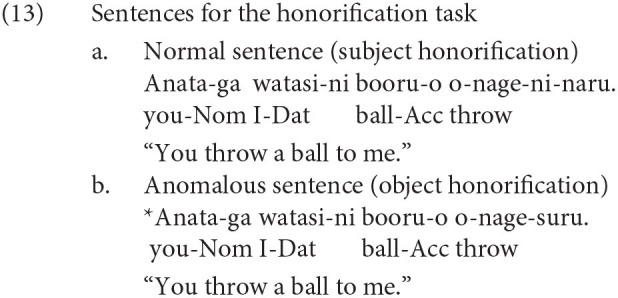



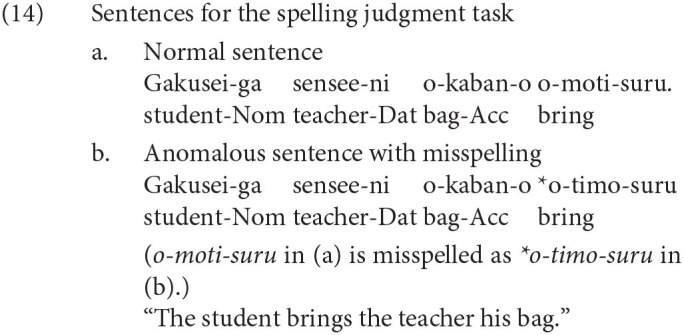


The stimulus sentences were visually presented to the participants as they were being scanned by an MRI scanner, and the participants were asked to judge whether each sentence was correct or incorrect by pushing one of two buttons. Momo et al. (2008) examined the brain regions in which the neural activation was greater in (13) than in (14). The correct judgment rates for (13) varied among the participants, and the neural activation patterns for (13) were correlated with judgments. Momo et al. (2008) observed greater activation in the left inferior frontal gyrus (LIFG) for (13) than for (14), especially in the participants whose correct judgment rates for (13) were relatively low. For the participants who often judged the honorific sentences in (13) correctly, a significantly greater activation against (14) was observed in the left lateral premotor cortex (LPMC) and the inferior parietal gyrus (and the cerebellum). According to Momo et al. (2008), the activation in the LIFG (including the left LPMC) for the honorification task suggested that syntactic computation for the agreement between the honorific verb and the subject or the object was involved in processing the honorification. We should note here, however, that the precuneus was reported as one of the regions that became significantly more activated by the honorification task than the spelling judgment task. Especially for participants who judged the honorific sentences correctly most of the time, the precuneus was the second most strongly activated region in the cerebral cortex. The precuneus is part of the theory of mind circuit (Mahy et al., 2014) and is deeply involved in perspective taking (Mizuno et al., 2011; Tokimoto and Tokimoto, 2018). It is not surprising to find significantly greater activation in the precuneus for the honorification task than for the spelling judgment task because the understanding of honorific expressions includes understanding the mental attitudes of the speaker toward the addressee. In this study, we attempted to examine the correspondence of our EEG results with the findings of Momo et al. (2008) by means of dipole fitting of the independent components (ICs) of the EEG and clustering of the ICs. EEG analysis is inferior to fMRI in terms of spatial resolution but is superior to fMRI in terms of temporal resolution.

Furthermore, we can analyze the event-related spectral perturbation (ERSP) to examine the spectral power for different frequency bands and analyze the intertrial phase coherence (ITC) for the difference between evoked and induced neural activities. The ERPs elicited by a violation of the linguistic constraint are often associated with the θ band (Roehm et al., 2004; Hald et al., 2006). However, Tokimoto and Tokimoto (2018) observed the suppression of the β band for perspective-taking in sentence comprehension. Understanding the human relationships in a sentence can involve perspective-taking of the persons described in the sentence, and thus, we can expect a contrast between honorific and control sentences in the β band. On the other hand, Tokimoto et al. (2019) observed a difference in the ITC increase in the frequency band depending on the type of syntactic processing, namely, an ITC increase in the θ band for the syntactic integration of discontinuous constituents and an ITI increase in the θ to γ bands for the violation of a syntactic island constraint. The research on linguistic ERSP and ITC is relatively new, but we can expect new findings regarding the frequency property specific to honorific processing, especially in the β band.

We enumerate our research questions in (15).

(15)     a.     If the social relationship between speakers is coded in subject-honorific and object-honorific expressions in Japanese, we can expect an N400 for anomalous sentences, as was observed in Jiang et al. (2013). On the other hand, if the regularity in the honorific expressions in Japanese is coded as grammatical rules, then a LAN is expected for the anomalous sentences. A P600 is also possible to be observed if the anomaly in Japanese honorific expressions is thematic.
            b.    If we accept the argument for the overly respectful use of *nin/nin-de* by Jiang et al. (2013), then late positivity is expected for the anomalous use of the subject-honorific verbs as a neural manifestation of the overly respectful use of the verbs. Furthermore, if we accept their argument for the disrespectful use of *ni/ni-de*, then late negativity is expected for the anomalous use of the object-honorific verbs as a manifestation of the disrespectful use of the verbs.
            c.    If our anomalous sentences elicit biphasic negative and positive ERPs, can a significant correlation in magnitude be found between them?
            d.    Can a significant difference in the neural activity in the left frontal region and/or in the medial part of the occipital region be found between subject-/object-honorific and control sentences and between appropriate and anomalous honorific sentences?
            e.    Can a significantly different neural activity be found in the frequency property (especially in the β band) between subject-/object-honorific and control sentences and between subject-honorific and object-honorific sentences?

## 2. Method

### 2.1. Participants

Eighteen native speakers of Japanese between 19 and 25 years of age (*M* = 21.67 years, *SD* = 1.37 years, 15 female) participated in this study for payment. They were undergraduate and graduate students. The participants had normal or corrected-to-normal vision and had no history of neurological/psychiatric disorders. All the participants were right-handed, as assessed by a handedness questionnaire (Oldfield, 1971).

This study was approved by the Ethics Committee of Mejiro University. Written informed consent was obtained from each participant.

### 2.2. Materials

The experimental sentences were simple sentences of four phrases with honorific verbs at the ends of the sentences. The experimental sentences were composed of four subject-honorific and four object-honorific verbs that describe four basic human behaviors: to see, to say, to go, and to know.

Their visual presentations, their numbers of characters, and their frequencies in Tsukuba Web Corpus are shown in Table 1, with their means and SDs in parentheses.

**Table 1:** Subject-honorific and object-honorific verbs included in the experimental sentences (pronunciations) and the number of characters in the visual presentation (SD) and their frequencies (SD) in Tsukuba Web Corpus (including approximately 1.1 billion words collected from Japanese websites).

**Meaning**	**Subject-honorific verb**	**Number of**	**Frequency**	**Object-honorific verb**	**Number of**	**Frequency**
		**characters**			**characters**	
saw	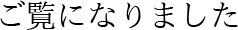	8	14,302	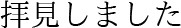	6	13,766
	(goraNni-narimashita)			(haiken-shimashita)		
said	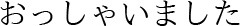	8	57,069	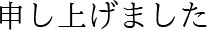	7	109,005
	(osshai-mashita)			(mooshiage-mashita)		
go	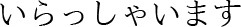	8	48,519	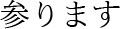	4	11,450
	(irasshai-masu)			(mairi-masu)		
know	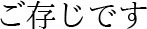	5	201	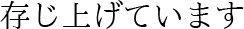	8	768
	(gozonzi-desu)			(zonziagete-imasu)		
Mean		7.25 (1.5)	30,023 (27,142)		7.25 (1.7)	33,747 (50,490)

To represent human relationships, two people were described in the experimental sentences, and they were placed in the first and third phrase. One person was represented by a title, that is, teacher, president, manager, senior managing director, or director-general, and the other person was the speaker ‘
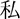
’ (I). The appropriate and anomalous sentences with the object-honorific verbs were variations in the subject-honorific sentences, composed by replacing the subject-honorific verbs with the corresponding object-honorific verbs, as presented in Table 1. That is, the appropriate object-honorific sentences exhibited variations in the anomalous subject-honorific sentences, and the anomalous object-honorific sentences exhibited variations in the appropriate subject-honorific sentences. Therefore, the three phrases preceding the verbs were identical between the subject-honorific and the object-honorific conditions. Some of the experimental sentences are presented in (16) to (19), in which the subject-honorific and object-honorific verbs are underlined.

Standard Japanese has three orthographic systems. Two of them are syllabaries, namely, *hiragana* and *katakana*, and the other is *kanji*, which is composed of historically Chinese characters. The stimulus sentences were written in the standard Japanese orthography, which is composed of all three orthographic systems. A subject-honorific sentence is appropriate when the subject is one of the titles above because the person with the title is naturally understood to be superior to the speaker. On the other hand, when the speaker ‘
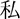
’ is placed as the subject of a subject-honorific verb, the sentence is anomalous. An object-honorific sentence is appropriate when the subject is the speaker ‘
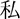
’ and the person with a title is included in the object or the adverbial argument because the person with a title can be naturally understood to be superior to the subject “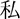.” Conversely, an object-honorific sentence is anomalous when the person with a title is the subject and the speaker is included in the object or the adverbial argument because the object-honorific verb requires the speaker “
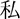
” to be superior to the person with a title.


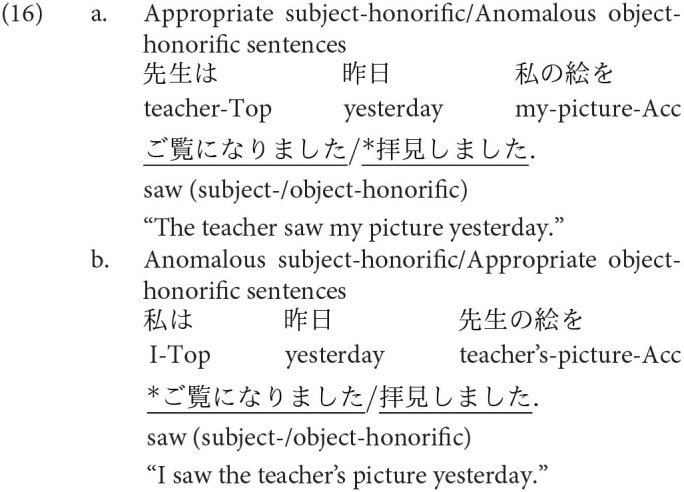



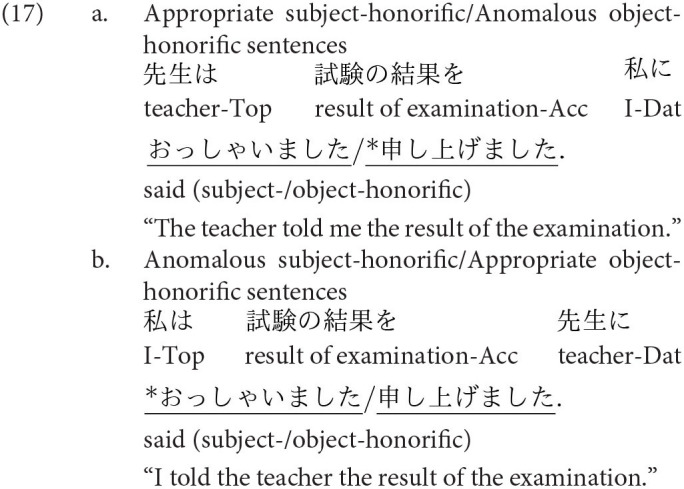



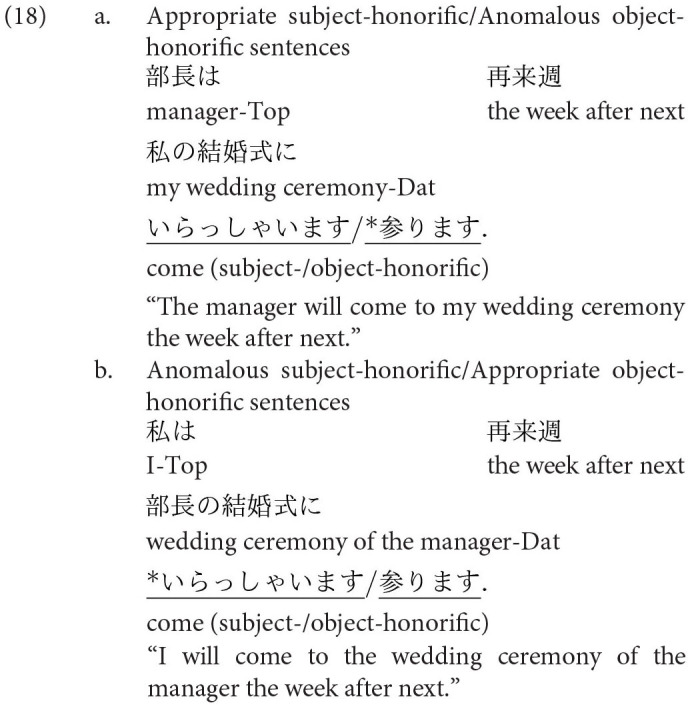



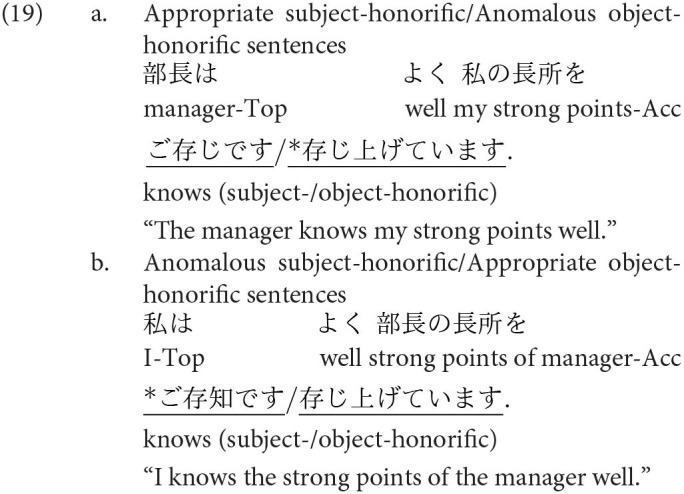


The person to be respected can be part of a complex noun phrase, as is shown in the appropriate object-honorific sentences (16-b), (18-b), and (19-b). Even when a person is part of a complex noun phrase, the sentence is judged as anomalous when an appropriate relationship between the two persons cannot be determined. The heads of the complex nouns genitively marked by “
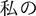
” (my) or the titles above were personality (career, biography, special qualifications, good point, ability), products (accomplishment, work, project proposal, graduation thesis), belongings (customer, daily duty, dream, car, photograph, painting, new house, resume), affiliation (department, office, hospital room, business trip destination), and meetings (farewell party, personal exhibition, debrief meeting, concert, wedding ceremony). Adverbials or accusative/dative objects that did not include human nouns were placed in the second phrases ^2^.

Forty sentences using an honorific verb were constructed, with 20 being appropriate and the other 20 being anomalous. Furthermore, the word order was changed in two ways so that the participants would not develop a strategy in the main session. That is, in half of the sentences, the first and the third phrases were exchanged so that the participants would not develop a strategy due to a monotonous sentence presentation. Forty simple sentences were also included in the main session as controls, with 20 being grammatical and the other 20 being ungrammatical. The ungrammatical sentences were ill-formed due to the wrong realization of the argument structure of a verb. Thirty of the controls included the speaker “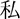,” in which fifteen were grammatical and the other fifteen were ungrammatical. Two of the controls are presented in (20)^3^.


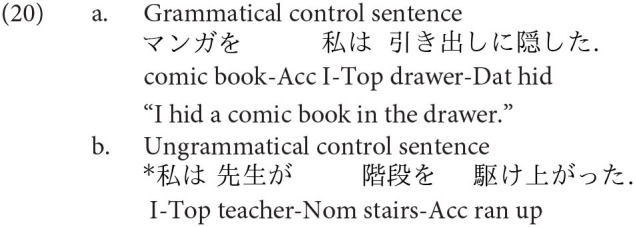


In total, three hundred and sixty sentences were randomly ordered and divided into four blocks.

### 2.3. Procedure

The participants were seated in an electrically and acoustically shielded EEG chamber 1 m in front of a 19-inch LCD monitor. Each trial began with the participant pressing a button press, and following the button press, a fixation point at the center of the monitor was shown for 1 s. A stimulus sentence was visually presented phrase by phrase after fixation at the center of the monitor with an SOA of 800 ms and an ISI of 100 ms. The presentation of each sentence was thus 4.2 s long. The participants were required to make acceptability judgments by pushing a button for each sentence (“acceptable” or “unacceptable”). The presentation order of the stimulus sentences was pseudo-randomized for each participant. The experiment was controlled using STIM2 software (Neuroscan). The practice session consisted of ten trials, the main session consisted of four blocks, and the participants were allowed to rest for 3 to 5 min between blocks. The experimental sessions, including instruction and the application of the electrodes, lasted approximately 2 h.

### 2.4. EEG Recording

EEG signals were recorded using a 32-channel EEG amplifier (NuAmps, Neuroscan) with an EasyCap electrode recording system (EasyCap; 10–20 montage) in which 21 electrodes were placed on the scalp (FP1, FP2, F7, F3, Fz, F4, F8, FCz, T7, C3, Cz, C4, T8, P7, P3, Pz, P4, P8, O1, Oz, O2). Vertical and horizontal electrooculograms (EOGs) were simultaneously recorded from electrodes below the left eye (vertical EOG: VEOG) and at the outer canthus of the right eye (horizontal EOG: HEOG). The signals were sampled at 250 Hz with a bandpass filter of 0.1–100 Hz with the reference electrodes positioned at the earlobes. The electrode impedance was maintained at a level lower than 10 kΩ during the sessions. EEG data were continuously acquired using SCAN4 software (Neuroscan).

### 2.5. EEG Data Preprocessing

The acquired EEG data were processed offline using EEGLAB (Delorme and Makeig, 2004) and ERPLAB (Luck, 2014). We analyzed the EEG data in two ways. The first analysis was intended to be for the direct comparison with Jiang et al. (2013), in which the EEGs on the scalp electrodes were referenced on line to the left mastoid and were rereferenced offline to the average of the activity at the left and the right mastoids and the baseline was corrected with the 200 ms prestimulus average EEG activity. The preprocesses of the first analysis proceeded as follows. (1) The data were high-pass filtered at 0.1 Hz (Hamming windowed FIR filter, order 826, bandwidth 1 Hz) with the reference of linked earlobes. (2) Line noise was removed using the CleanLine plugin in EEGLAB. (3) High-amplitude artifacts were removed from the EEG data using the artifact subspace reconstruction (Mullen et al., 2015). (4) The data were decomposed using an adaptive mixture of independent component analyzers (AMICA) (Palmer et al., 2007). (5) The best-fitting single equivalent current dipole was calculated for each IC to match the scalp projection of each IC source using a standardized three-shell boundary element head model. The electrode locations were aligned corresponding to the 10–20 system with a standard brain model (Montreal Neurological Institute). (6) The possible sources were evaluated for each IC with the ICLabel plugin in EEGLAB as follows (Pion-Tonachini et al., 2017): brain neural activity, EOG, muscle potentials, electrocardiogram, line noise, channel noise, and others. We chose the ICs for which the possibility of brain neural activity percentages were greater than 70% for the following analyses. (7) The ICs were excluded from further analysis for instances in which the equivalent dipole model explained less than 85% of the variance in the corresponding IC scalp map. The percentage of remaining ICs ranged from 30.4 to 60%, with an average of 49.9%. (8) The EEG data were segmented into time epochs relative to event markers from −1 to 2 s from the markers. The epochs in which EEE or EOG exceeded ± 70 μV were removed from the following analyses, and the percentage of the removed epochs was 4.82%. In this first analysis, the EEG data was analyzed with a prestimulus baseline of 100 ms, which corresponded to the ISI of the stimulus presentation.

In the second analysis, the EEG data were high-pass filtered at 1 Hz (EEGLAB Basic FIR Filter New, zero-phase, finite impulse response, −6 dB cutoff frequency 0.5 Hz, transition bandwidth 1 Hz) as the first preprocessing step because high-pass filtering between 1 and 2 Hz was reported to produce good results in terms of signal-to-noise ratio as a preprocessing step for ICA-based artifact removal (Winkler et al., 2015). After the artifact removal by the artifact subspace reconstruction, the EEG data were rereferenced to a common average reference as the fourth step. This is because the physical head model assumes a zero integral over the head surface, and thus the average reference removes the bias of the reference electrode(s). Furthermore, since the EEG signal was assumed to become cleaner as the noise common to all the electrodes got subtracted by average referencing, a small ERP component could be detected. The preprocesses from the IC decomposition by AMICA to the epoch segmentation were the same with the first analysis. In the second analysis, the percentage of remaining ICs ranged among the participants from 38.1 to 63.6%, with the average as 53.3%, and the percentage of the removed epochs in which EEE or EOG exceeded ± 70 μV was 0.4%. In the second analysis, the EEG data was analyzed with a poststimulus baseline of 100 ms. When we examined the contrast between the appropriate and the anomalous subject- or object-honorific sentences at the honorific verbs, the sequences of preceding words were different between the appropriate and the anomalous conditions. We cannot assume therefore the same neural activities right before the verbs for the two conditions. A linguistic ERP appears in a relatively late time window compared to visual evoked potentials, which appear earlier than the latency of 200 ms most of the time. Our main concern was the neural activity expected to be a manifestation of the higher processing of human relationships, and therefore we assumed that the neural activities at the same verb could be effective to detect a late ERP component for the higher processing. This was the reason that we analyzed the EEG data with a poststimulus baseline in the second analysis^4^.

## 3. Results

### 3.1. Behavioral Results: Acceptability Judgments

**Figure 1:**
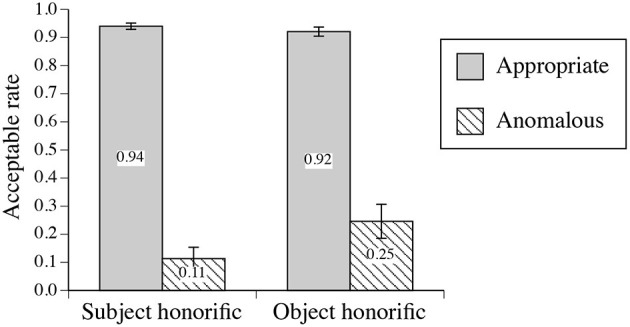
Mean acceptable judgment rates of the experimental sentences for the two types of honorific expressions and appropriateness (appropriate or anomalous). The error bars indicate the standard errors.

Figure 1 presents the mean proportions of acceptable judgments for the two honorific (subject-honorific and object-honorific) expressions and for appropriateness (appropriate or anomalous).

A two-way ANOVA was conducted for the acceptable rates with the two types of honorific expressions (subject-honorific or object-honorific) and appropriateness (appropriate or anomalous) as within factors. The main effect of the type of honorific expression was significant [$F_{\left(1,\;17\right)}=6.83,MSe=0.008,p=0.018,\eta_{p}^{2}=0.29$], the main effect of appropriateness was significant [$F_{\left(1,\;17\right)}=209.91,MSe=0.048,p<0.001,\eta_{p}^{2}=0.93$], and the interaction between the type of honorific expression and appropriateness was significant [$F_{\left(1,\;17\right)}=8.82,MSe=0.012,p=0.009,\eta_{p}^{2}=0.34$]. Tukey's HSD test indicated that the acceptable rate for the anomalous object-honorific sentences was marginally significantly greater than that for the anomalous subject-honorific sentences [*p* = 0.071].

### 3.2. ERP Analysis

To examine the neural effect of honorific expressions, we analyzed the ERP time-locked to the onset of the honorific verbs in the fourth phrases and examined the contrast between appropriate and anomalous sentences. We analyzed the ERP with a prestimulus baseline of 100 ms for the first analysis and with a poststimulus baseline of 100 ms for the second analysis, as was discussed in the subsection of EEG data preprocessing.

In the first analysis, we mainly examined the contrast between the appropriate and the anomalous conditions in the standard time windows of LAN/N400 (300–500 ms) and of P600 (500–800 ms) for the direct comparisons of our results with those of Jiang et al. (2013).

In the second analysis, we calculated the mean ERPs elicited by appropriate and anomalous verbs every 50 ms from 100 to 900 ms of latency and chose the electrodes at which the cluster-based permutation test indicated that there was a significant difference between the two conditions (Maris and Oostenveld, 2007). We then calculated the mean waveforms of the electrodes chosen for the two conditions and evaluated the approximate time window during which the difference between the two mean waveforms was significant by cluster-based permutation tests. The comparison between the two conditions was corrected by cluster-based permutation tests. The analyses of the condition effects in ERP were performed using the STUDY command structure in EEGLAB. Non-parametric random permutation statistics were computed to test the significance of the condition effects. In the current study, we computed 2,000 random permutations and compared them to the *t*-values for the mean condition differences.

#### 3.2.1. ERP at Verbs of Control Sentences

**Figure 2:**
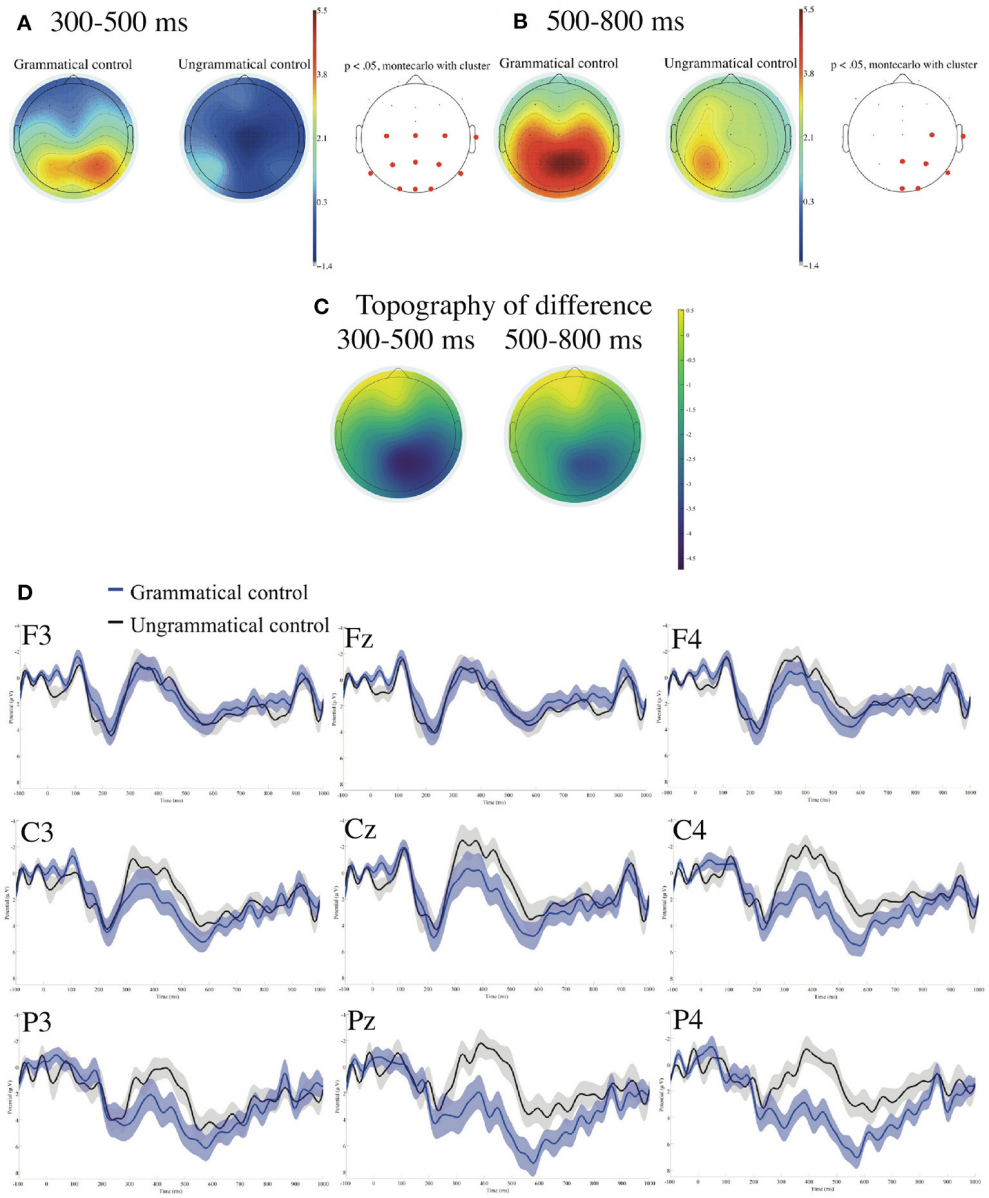
ERPs time-locked to the onsets of the verbs in control sentences **(A–D)** referenced to the linked earlobes with the prestimulus baseline from −100 to 0 ms. **(A)** Mean topographies of the ERPs from 300 to 500 ms, **(B)** those from 500 to 800 ms, and **(C)** the mean topographies of the difference in ERP amplitudes, for which we subtracted the amplitudes for the grammatical condition from those for the ungrammatical condition for the time windows of 300–500 and 500–800 ms, respectively. The electrode sites at which significant differences were found using the cluster-based permutation test (*p* < 0.05) are depicted in red. **(D)** ERP waveforms at nine electrodes from −100 to 1,000 ms for the grammatical and ungrammatical control sentences with the standard errors. Negativity is plotted upward.

Figure 2 presents ERPs time-locked to the onsets of the verbs in control sentences referenced to the linked earlobes with the prestimulus baseline from −100 to 0 ms. We found significant negative deflections for the ungrammatical against the grammatical controls in the (right) centro-parietal region in the time window of 300–500 ms and in the time window of 500–800 ms.

#### 3.2.2. ERP at Subject-Honorific Verbs

##### 3.2.2.1. First Analysis for Subject-Honorific Verbs

**Figure 3:**
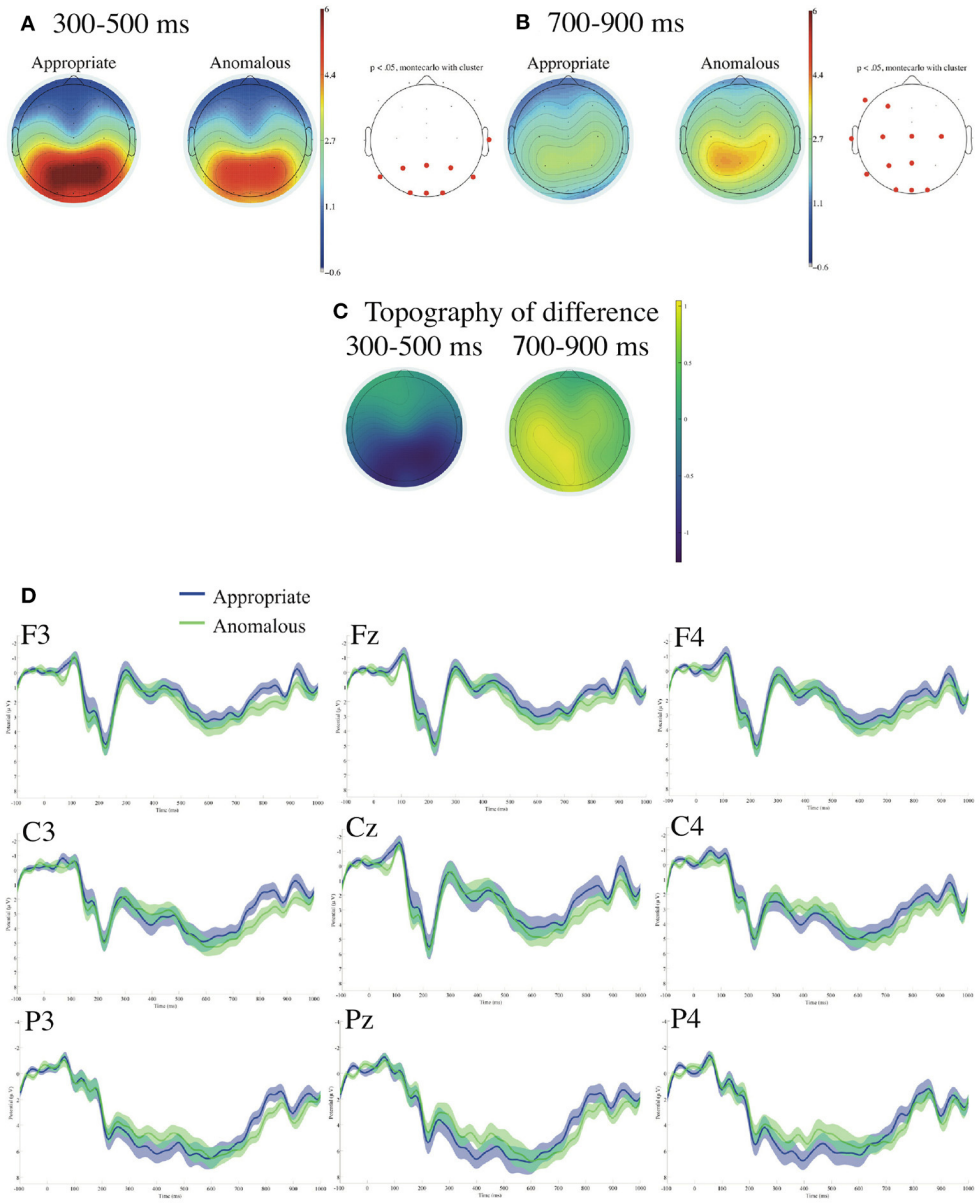
ERPs time-locked to the onsets of the subject-honorific verbs referenced to the linked earlobes **(A–D)** with the prestimulus baseline from −100 to 0 ms. **(A)** Mean topographies of the ERPs from 300 to 500 ms, **(B)** those from 700 to 900 ms, and **(C)** the mean topographies of the difference in ERP amplitudes, for which we subtracted the amplitudes for the appropriate condition from those for the anomalous condition for the time windows of 300–500 and 700–900 ms. The electrode sites at which significant differences were found using the cluster-based permutation test (*p* < 0.05) are depicted in red. **(D)** ERP waveforms at nine electrodes from −100 to 1,000 ms for appropriate and anomalous sentences, with the standard errors. Negativity is plotted upward.

Figure 3 presents ERPs time-locked to the onsets of the subject-honorific verbs referenced to the linked earlobes with the prestimulus baseline from −100 to 0 ms. We found a significant negative deflection for the anomalous against the appropriate sentences in the parieto-occipital region in the standard time window of N400 (300–500 ms), and we found no significant deflection in the standard time window of P600 (500–800 ms). We also found a significant positive deflection for the anomalous against the appropriate sentences in the left centroparietal region in the time window of 700–900 ms by the calculations of the mean ERPs for every 50 ms from 0 to 900 ms. No significant difference was observed at the two EOG electrodes (VEOG and HEOG) in the time window of 0–1,000 ms.

##### 3.2.2.2. Second Analysis for Subject-Honorific Verbs

**Figure 4:**
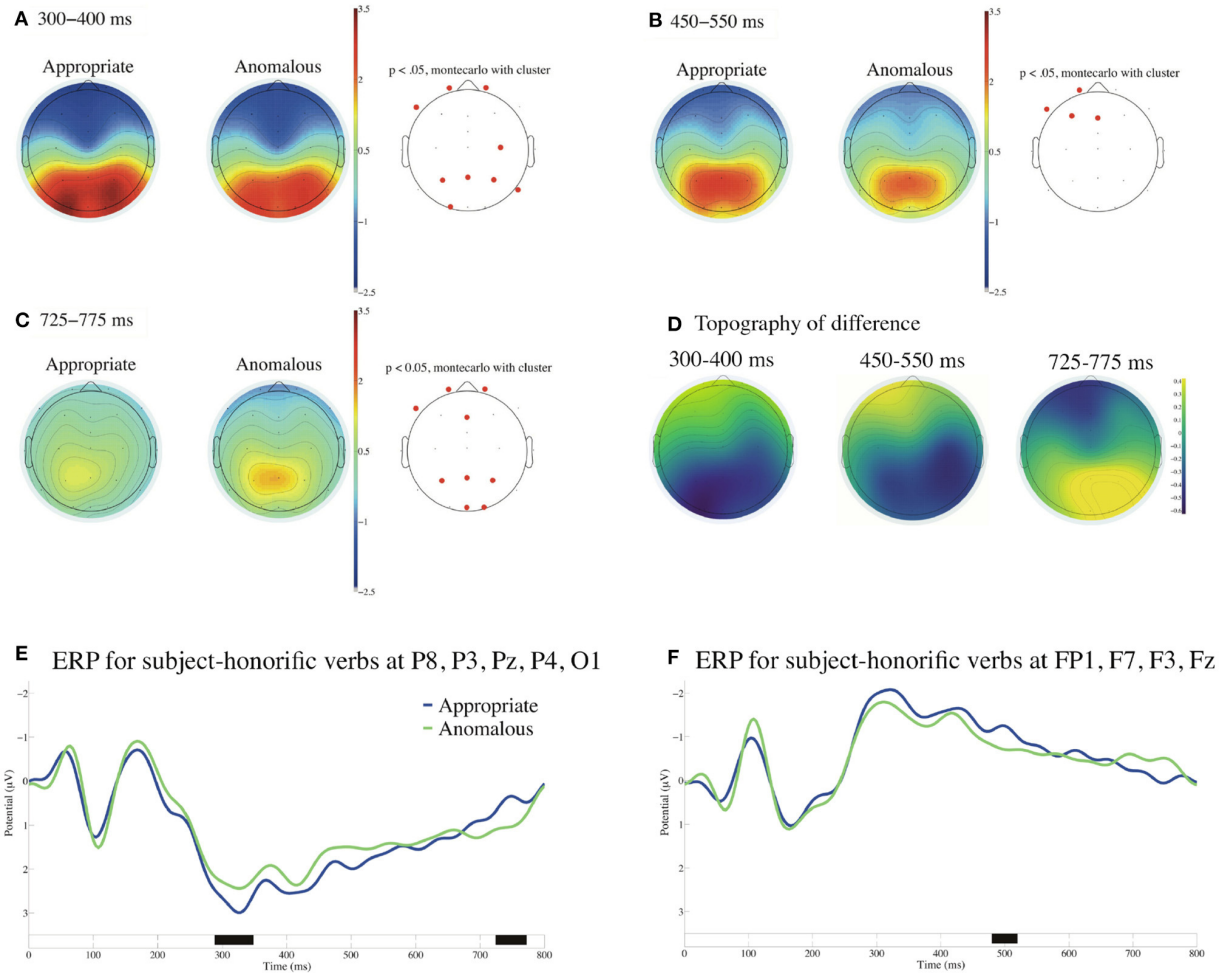
ERPs time-locked to the onsets of the subject-honorific verbs in **(A–F)** with the average reference and with the poststimulus baseline from 0 to 100 ms. **(A)** Mean topographies of the ERPs from 300 to 400 ms, **(B)** those from 450 to 550 ms, and **(C)** those from 725 to 775 ms for appropriate and anomalous conditions. The electrode sites at which significant differences were found using the cluster-based permutation test (*p* < 0.05) are depicted in red (Maris and Oostenveld, 2007). **(D)** Mean topographies of the difference in ERP amplitudes, for which we subtracted the amplitudes for the appropriate condition from those for the anomalous condition for the time windows of 300–400, 450–550, and 725–775 ms. **(E)** Mean ERPs of the two conditions at the parieto-occipital electrodes (P8, P3, Pz, P4, and O1) and **(F)** Mean ERPs of the two conditions at the left frontal electrodes (FP1, F7, F3, and Fz). Negativity is plotted upward, and the approximate temporal extensions of the significant clusters are indicated in black on the time axis in **(E,F)**.

Figure 4 presents the ERP contrasts at subject-honorific verbs between the appropriate and the anomalous sentences with the average reference and with the poststimulus baseline of 0 to 100 ms. The cluster-based permutation test indicated that there was a significant difference between appropriate and anomalous subject-honorific sentences. As Figure 4E shows, a cluster of significant negativity for the anomalous sentences was observed in the parietal region and extended from approximately 290 to 350 ms (Sassenhagen and Draschkow, 2019). As Figures 4E,F show, on the other hand, a cluster of significant positivity for the anomalous sentences was observed in the frontal region and extended from approximately 480 to 520 ms, and another cluster of significant positivity for the anomalous sentences was observed in the parietal region and extended from approximately 730 to 770 ms. No significant difference was observed at the two EOG electrodes in the time window of 100–1,000 ms.

#### 3.2.3. ERP at Object-Honorific Verbs

##### 3.2.3.1. First Analysis for Object-Honorific Verbs

**Figure 5:**
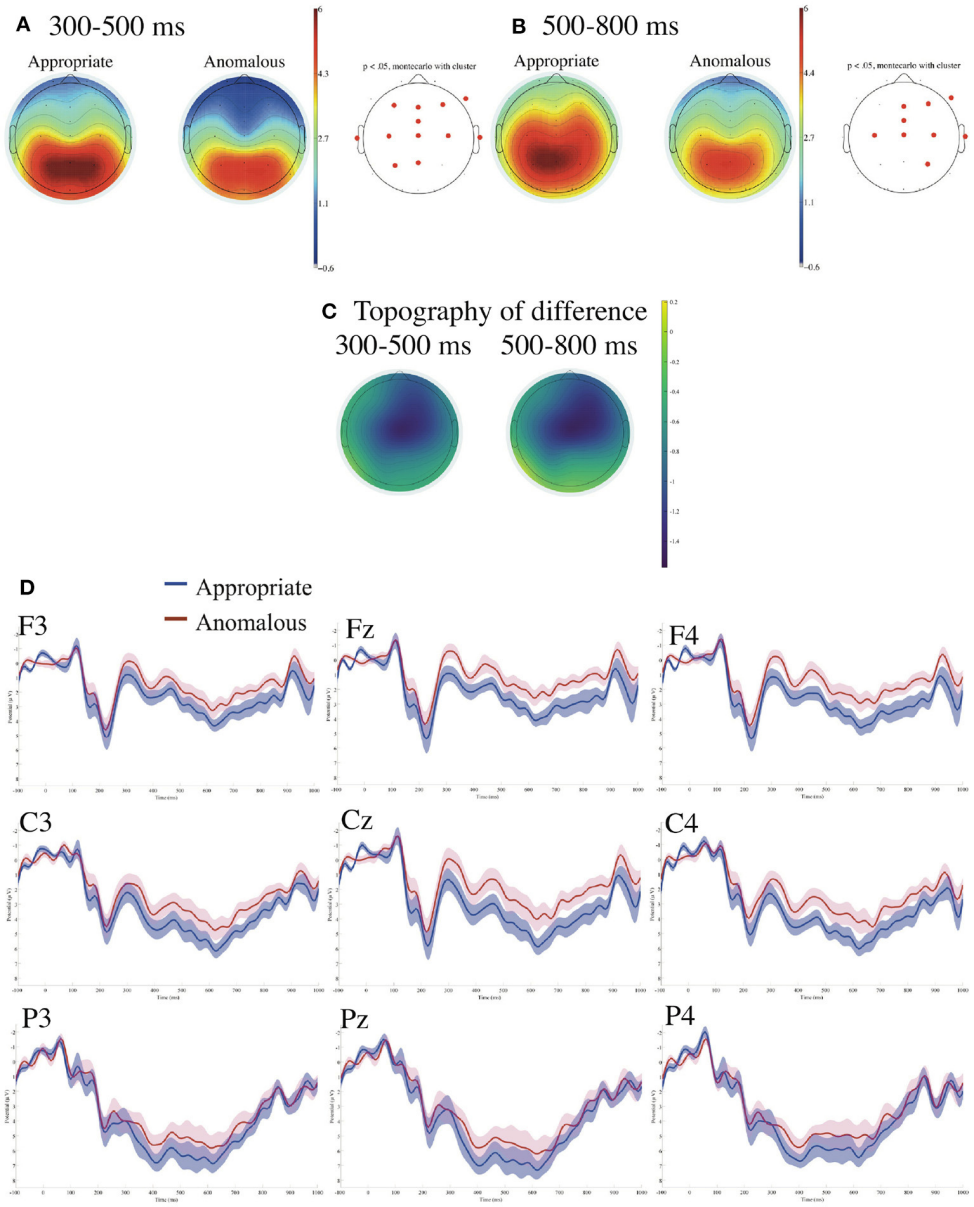
ERPs time-locked to the onsets of the object-honorific verbs referenced to the linked earlobes for the appropriate and the anomalous sentences **(A–D)** with the prestimulus baseline from −100 to 0 ms. **(A)** Mean topographies of the ERPs from 300 to 500 ms, **(B)** those from 500 to 800 ms, and **(C)** the mean topographies of the difference in ERP amplitudes, for which we subtracted the amplitudes for the appropriate condition from those for the anomalous condition for the time windows of 300–500 and 500-800 ms. The electrode sites at which significant differences were found using the cluster-based permutation test (*p* < 0.05) are depicted in red. **(D)** ERP waveforms at nine electrodes from −100 to 1,000 ms for appropriate and anomalous sentences with the standard errors. Negativity is plotted upward.

Figure 5 presents ERPs time-locked to the onsets of the object-honorific verbs referenced to the linked earlobes and with the baseline from −100 to 0 ms for the appropriate and the anomalous conditions. We can find significant negative deflections for the anomalous against the appropriate sentences in the frontocentral region in the standard time window of N400 (300–500 ms) and also in the standard time window of P600 (500–800 ms). No significant difference was observed at the two EOG electrodes in the time window of 0–1,000 ms.

##### 3.2.3.2. Second Analysis for Object-Honorific Verbs

**Figure 6:**
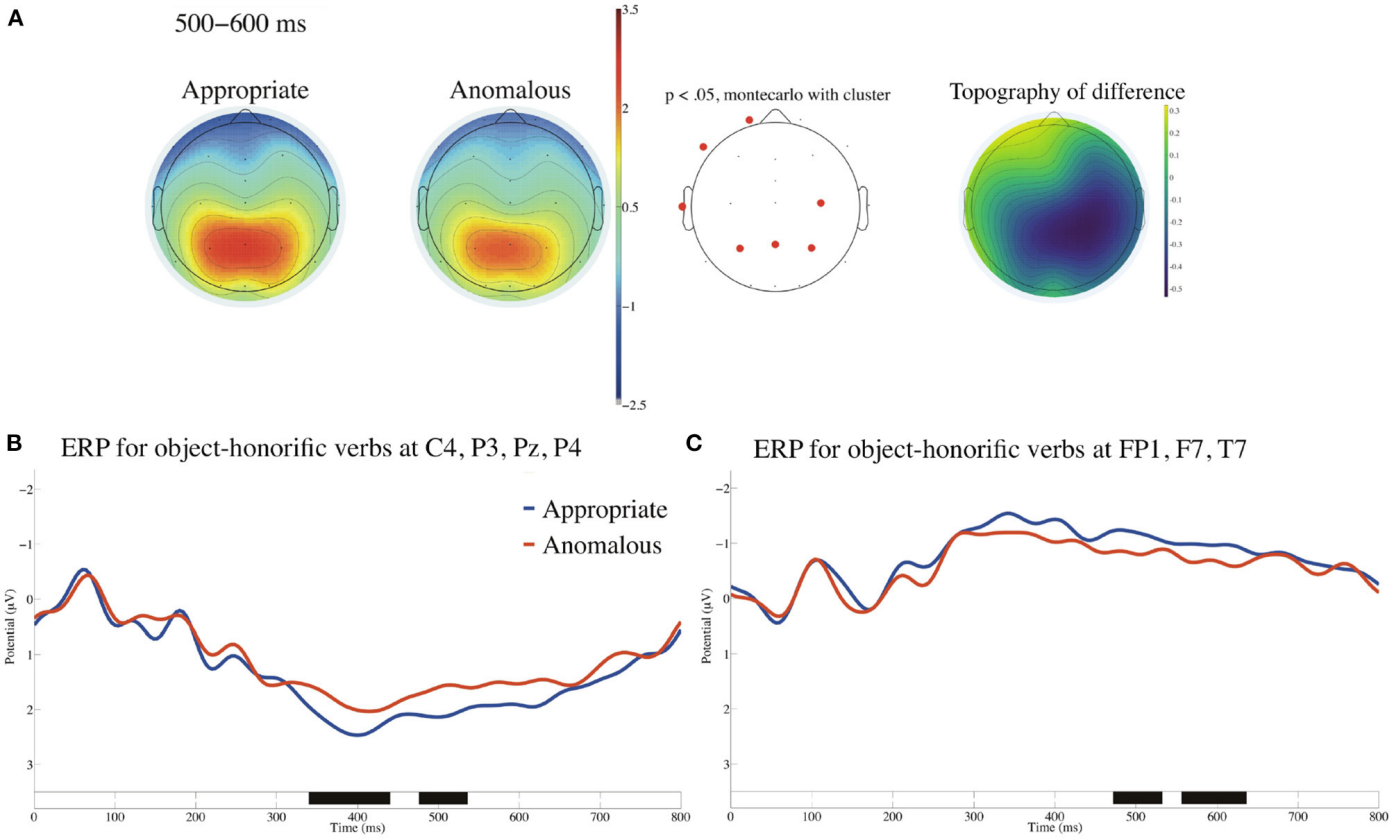
ERPs time-locked to the onsets of the object-honorific verbs in **(A–C)** with the average reference and with the poststimulus baseline from 0 to 100 ms. **(A)** Mean topographies of the ERPs from 500 to 600 ms for the appropriate and the anomalous conditions. The electrode sites at which significant differences were found using the cluster-based permutation test (*p* < 0.05) are depicted in red. The mean topography of the difference in ERP amplitudes, for which we subtracted the amplitudes for the appropriate condition from those for the anomalous condition for the time windows 500–600 ms. **(B)** Mean ERPs of the two conditions at the parietal electrodes (C4, P3, Pz, and P4). **(C)** Mean ERPs of the two conditions at the left temporo-frontal electrodes (FP1, F7, and T7). Negativity is plotted upward, and the approximate temporal extensions of the significant clusters are indicated in black on the time axis in **(B,C)**.

Figure 6 presents the ERPs time-locked to the onsets of the object-honorific verbs with the average reference and with the poststimulus baseline from 0 to 100 ms. The cluster-based permutation test indicated that there was a significant difference between appropriate and anomalous object-honorific sentences. A cluster of significant negativity for the anomalous sentences was observed in the parietal region and extended from approximately 330 to 440 ms and from 480 to 530 ms, as shown in Figure 6B. A cluster of significant positivity for the anomalous sentences was observed in the frontal region and extended from approximately 470 to 530 ms and from 550 to 640 ms, as shown in Figure 6C. No significant difference was observed at two EOG electrodes in the time window of 100–1,000 ms.

#### 3.2.4. ERP Contrast Between Subject- and Object-Honorific Verbs

The appropriate and the anomalous experimental sentences were made by replacing the subject-honorific verb with the corresponding object-honorific verb and vice versa. Therefore, it would be effective to examine the ERP contrast between the anomalously used subject-honorific and the appropriately used object-honorific verbs and the contrast between the anomalously used object-honorific and the appropriately subject-honorific verbs with a prestimulus baseline. This is because we could assume the same neural activity right before the verbs for the two conditions since the word sequences from the first to the third phrases were the same for them.

**Figure 7:**
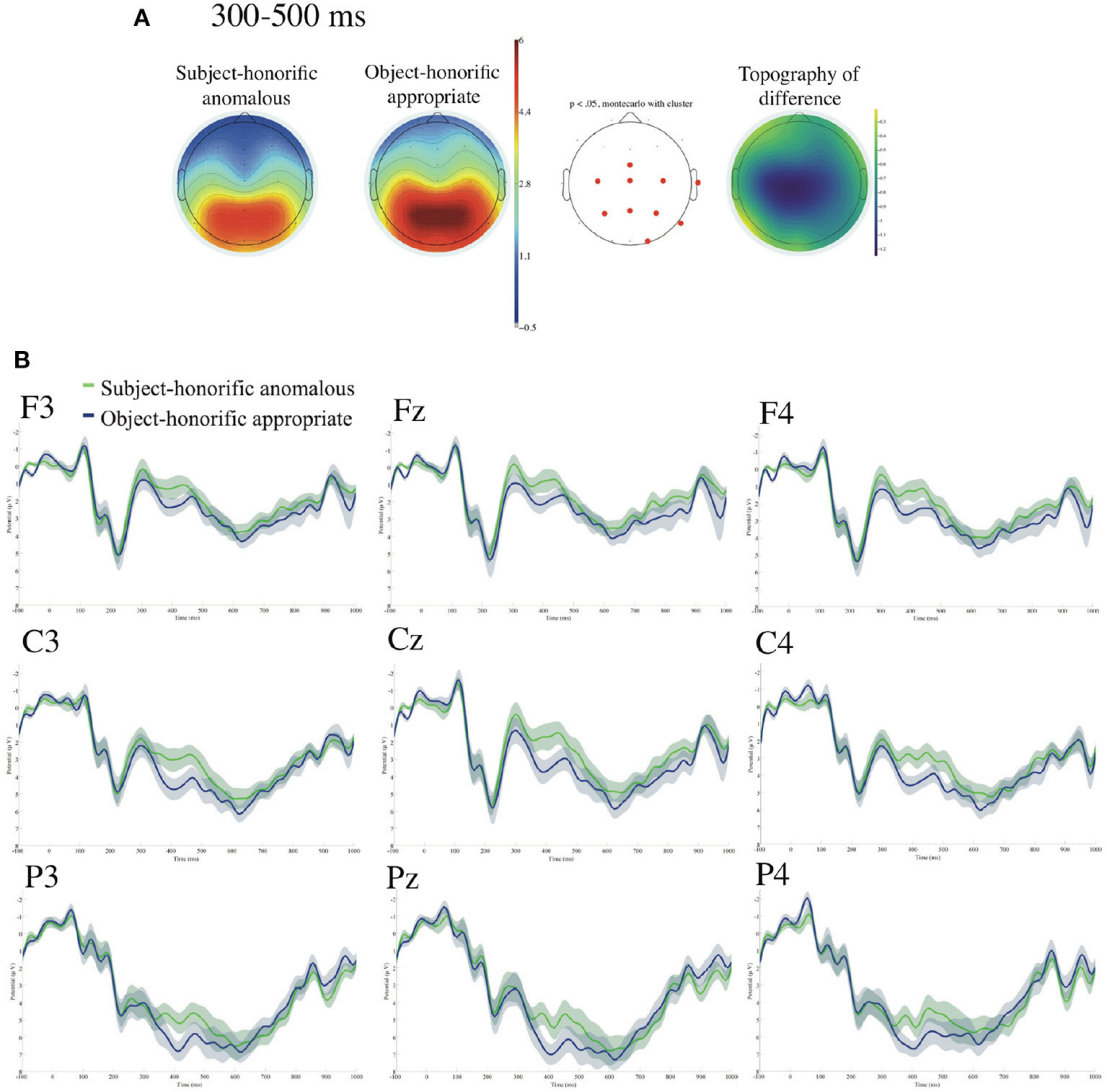
ERPs time-locked to the onsets of the anomalous subject-honorific and the appropriate object-honorific verbs referenced to the linked earlobes **(A,B)** with the prestimulus baseline from −100 to 0 ms. **(A)** Mean topographies of the ERPs from 300 to 500 ms, those from 500 to 800 ms, and the mean topography of the difference in ERP amplitudes, for which we subtracted the amplitudes for the appropriate object-honorific condition from those for the anomalous subject-honorific condition for the time windows of 300–500 ms. The electrode sites at which significant differences were found using the cluster-based permutation test (*p* < 0.05) are depicted in red. **(B)** ERP waveforms at nine electrodes from −100 to 1,000 ms for appropriate and anomalous sentences, with the standard errors. Negativity is plotted upward.

Figure 7 presents ERPs time-locked to the onsets of the anomalously used subject-honorific verbs and the appropriately used object-honorific verbs referenced to the linked earlobes with the prestimulus baseline from -100 to 0 ms. We find significant negative deflections for the anomalous subject-honorific against the appropriate object-honorific sentences in the centroparietal region in the standard time window of N400 (300–500 ms), and we find no significant deflection in the standard time window of P600 (500–800 ms). No significant difference was observed at two EOG electrodes in the time window of 0–1,000 ms.

**Figure 8:**
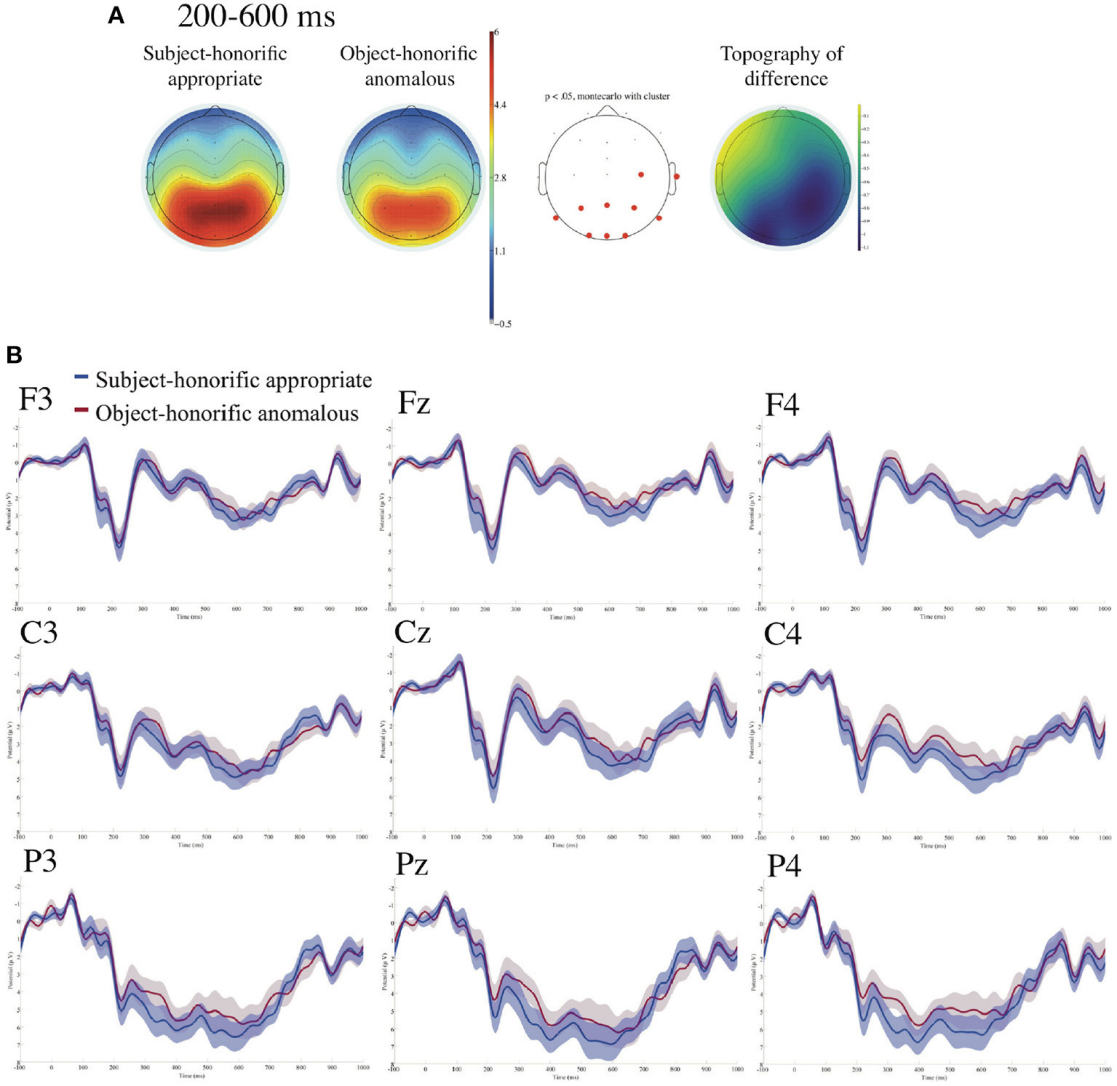
ERPs time-locked to the onsets of the appropriate subject-honorific and the anomalous object-honorific verbs referenced to the linked earlobes **(A,B)** with the prestimulus baseline from −100 to 0 ms. **(A)** Mean topographies of the ERPs from 200 to 600 ms and the mean topography of the difference in ERP amplitudes, for which we subtracted the amplitudes for the appropriate subject-honorific condition from those for the anomalous object-honorific condition for the time windows of 200–600 ms. The electrode sites at which significant differences were found using the cluster-based permutation test (*p* < 0.05) are depicted in red. **(B)** ERP waveforms at nine electrodes from −100 to 1,000 ms for appropriate and anomalous sentences, with the standard errors. Negativity is plotted upward.

Figure 8 presents ERPs time-locked to the onsets of the appropriately used subject-honorific verbs and the anomalously used object-honorific verbs referenced to the linked earlobes with the prestimulus baseline from −100 to 0 ms. We find no significant contrast between the two conditions for the standard time windows of N400 and of P600. However, we find a significant negative deflection for the anomalous object-honorific against the appropriate subject-honorific condition in the right centroparietal region in the time window of 200–600 ms by the calculations of the mean ERPs for every 50 ms from 0 to 900 ms.

### 3.3. Correlations Between Negative and Positive ERPs

In this section, we discuss the correlations between the negative and the positive ERPs found for subject-honorific verbs in the contrast between the appropriate and the anomalous sentences to examine the possible interactions between them, keeping in mind the recent finding that a negative ERP counteracted a positive ERP elicited by semantic anomalies (Kim et al., 2018) and the finding that a negative ERP enhanced the following positive ERP in syntactic processing (Tokimoto et al., 2019).

#### 3.3.1. Correlations in Subject-Honorific Verbs

Table 2 presents the correlation between the mean amplitude of the negative ERP and that of the positive ERP and between the maximum amplitude of the negative ERP and that of the positive ERP in the time windows in which the contrasts between the appropriate and anomalous sentences for the subject-honorific verbs were significant in the second analysis in Figures 4E,F.

**Table 2:** Means of the mean amplitudes and the maximum amplitudes of the ERPs for the anomalously used subject-honorific verbs in the fourth phrases, with *r* as the correlation coefficient.

**Subject-honorific verbs**	**Negative ERP**	**Positive ERP**	**r**
Electrodes	P8, P3, Pz, P4, O1	FP1, F7, F3, Fz	
Time window (ms)	280–350	480–520	
Mean of the mean amplitudes (μV, SD)	1.89 (1.05)	−1.05 (0.70)	−0.55^*^
Mean of the maximum amplitudes (μV, SD)	1.44 (1.05)	−0.81 (0.71)	−0.68^**^

* p < 0.05,

** *p < 0.01*.

The correlations between the negative ERP and the positive ERP were significantly negative for the mean amplitudes and the maximum amplitudes. The amplitudes of positivity were negative; thus, the negative correlation coefficients indicate that a reader with a greater negative ERP magnitude showed a greater positive ERP magnitude. Therefore, the preceding negativity could enhance the following positivity for the subject-honorific verbs. However, no significant correlation in amplitude was found between the negativity (300–500 ms) and the positivity (700–900 ms) for the first analysis, which was shown in Figures 3A,B.

As for the object-honorific verbs, we found a significant positivity only in the second analysis, and therefore we did not examine the correlation between the negative and the positive deflections.

When using an average electrode reference, the voltages of all electrodes across the scalp must, by definition, sum to zero at each time point. This means that for every local negativity or positivity, there will be a corresponding voltage of opposite polarity created elsewhere on the scalp. This approach may have produced the significant frontal positivity that we found in the second analysis for both subject and object honorific anomalous sentences.

### 3.4. IC Cluster Analysis

In this section, we discuss the correspondence of our EEG data to the findings of Momo et al. (2008). We clustered the ICs that remained in the preprocessing for the second analysis into three clusters for cluster analysis using a k-means algorithm for the dipole locations of the ICs. The number of clusters was determined by two criteria: the number of participants in each cluster and the silhouette values (Rousseeuw, 1987). As the first step, we attempted to construct as many clusters as possible to the extent that all the participants were included in a cluster, and we found that four clusters always included all the participants in the 20 clustering repetitions. We then calculated the average silhouette values of all the ICs corresponding to the number of clusters (2–4). The silhouette value indicates the degree of confidence that a certain IC belongs to a certain cluster and not to the other clusters. The silhouette value is 1 when all members of a cluster are identical (best separation), and it is −1 in the worst case. The remaining epoched ICs were clustered into three clusters because we obtained the greatest average silhouette value for three clusters.

**Figure 9:**
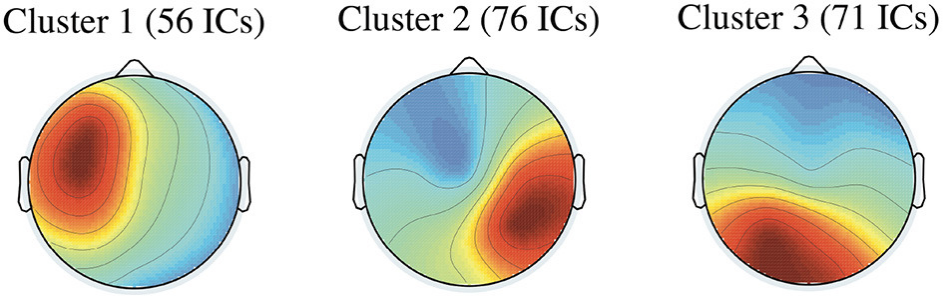
Average scalp maps for the three IC clusters (number of ICs).

Figure 9 presents the average scalp maps of the three IC clusters, with the number of ICs in parentheses.

The number of ICs of each participant for the three IC clusters are presented in Table 3.

**Table 3:** The number of ICs of each participant for the three IC clusters.

**Participant number**	**1**	**2**	**3**	**4**	**5**	**6**	**7**	**8**	**9**	**10**	**11**	**12**	**13**	**14**	**15**	**16**	**17**	**18**	**Total**
Cluster 1	3	4	3	2	3	4	1	4	2	3	5	3	4	3	4	4	2	2	56
Cluster 2	4	2	5	3	4	3	5	4	5	4	3	5	4	6	5	2	7	5	76
Cluster 3	4	4	4	4	3	5	5	4	2	4	4	3	6	4	5	2	5	3	71

Table 4 summarizes the following properties of the three IC clusters: the numbers of ICs, the mean coordinates in Talairach space (Talairach and Tournoux, 1988), the mean residual variances (r.v.), the hemispheres, the structures, and the Brodmann area (BA) corresponding to the mean coordinates.

**Table 4:** The numbers of ICs included in the three clusters, their mean coordinates in Talairach space, their mean residual variances (r.v.), their hemisphere, their structure, and the Brodmann area (BA) corresponding to their mean coordinates.

	**Number of ICs**	**Mean coordinate**	**Mean r.v. (%)**	**Hemisphere**	**Structure**	**BA**
Cluster 1	56	−29, 10, 28	4.27	Left	Precentral gyrus	6
Cluster 2	76	38, −15, 26	4.31	Right	Precentral gyrus	6
Cluster 3	71	−15, −68, 17	2.91	Left	Cuneus	18

**Figure 10:**
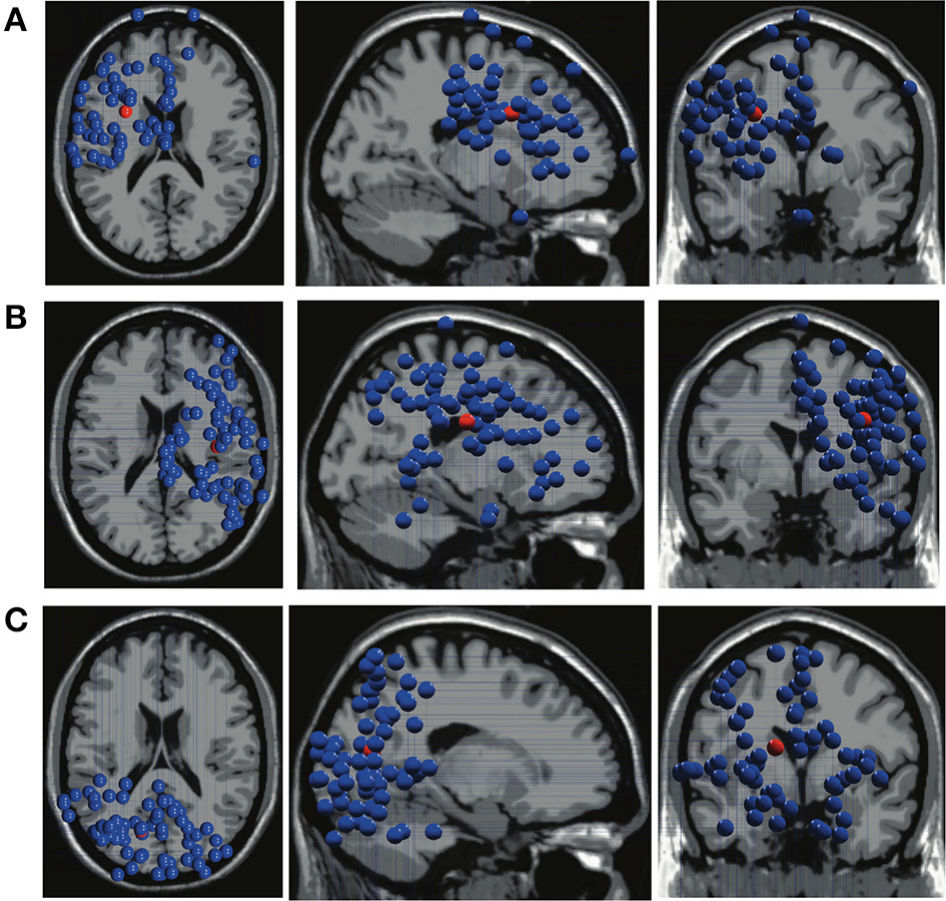
The dipole locations of Cluster 1 in **(A)**, Cluster 2 in **(B)**, and Cluster 3 in **(C)**. The top, sagittal, and coronal views from left to right. The red balls in the dipole locations indicate the mean coordinate positions.

The dipole locations of Clusters 1, 2, and 3 are presented in Figure 10.

#### 3.4.1. IC Cluster Analysis for Honorific Verbs and Control Verbs

Momo et al. (2008) examined the neural activity in the honorification task compared to that in the spelling judgment task. The subject or object of their honorific sentence agreed with the honorific verb, and the main concern of Momo et al. (2008) was the presence or absence of syntactic processing associated with the agreement between an honorific verb and the subject or the object. The normal sentence for subject honorification in Momo et al. (2008) in (13-a) corresponds to our appropriate subject-honorific sentence, whereas the anomalous sentence for object honorification in (13-b) corresponds to our anomalous object-honorific sentence. To compare our EEG results with the fMRI findings of Momo et al. (2008), we analyzed the ERP time-locked to the two types of honorific verbs as one condition and compared it with the ERP time-locked for the verbs in the control sentences.

**Figure 11:**
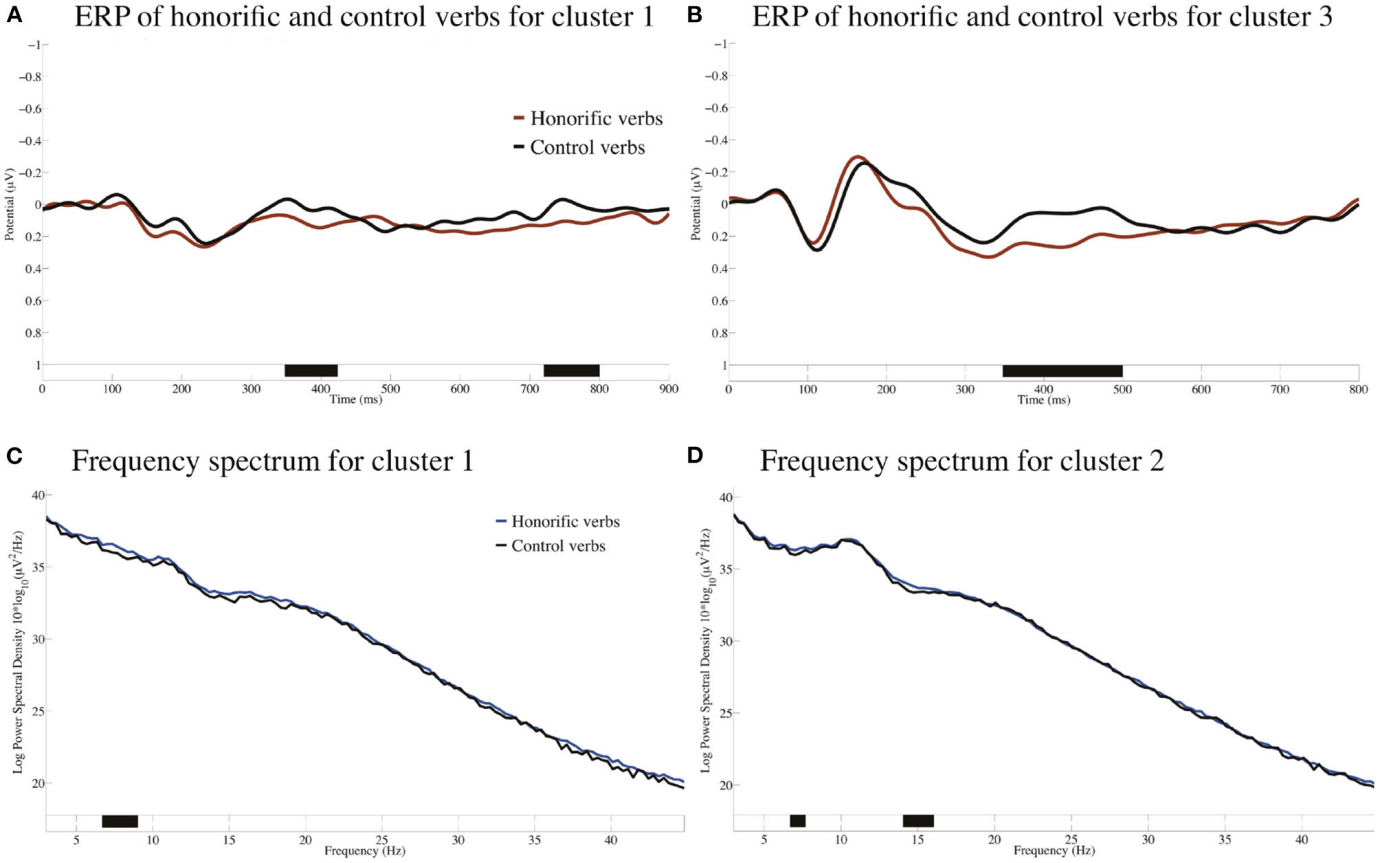
ERPs for Cluster 1 in **(A)** and Cluster 3 in **(B)** time-locked to the onsets of the honorific verbs and the control verbs with the baseline from 0 to 100 ms. Negativity is plotted upward, and the approximate temporal extensions of the significant clusters (*p* < 0.05) are indicated in black on the time axis in **(A,B)**. Power spectral densities for the honorific and control verbs for Cluster 1 **(C)** and for Cluster 2 **(D)**. The approximate frequency extensions of the significant clusters are indicated in black on the frequency axis in **(C,D)**.

Figure 11 shows the ERPs of Cluster 1 and Cluster 3 time-locked to the onsets of the honorific verbs and the control verbs with the poststimulus baseline from 0 to 100 ms and the frequency spectra for Cluster 1 and Cluster 2.

The cluster-based permutation test indicated that there was a significant difference in ERP between the honorific and control verbs in Cluster 1 and Cluster 3. A cluster of significant positivity for the honorific verbs against the control verbs extended from approximately 350 to 430 ms and from 720 to 800 ms in Cluster 1 and 350–500 ms in Cluster 3. As for the frequency spectra, the cluster-based permutation test indicated that there was a significant difference between the honorific and control verbs in Cluster 1 and Cluster 2. A cluster of significantly greater power spectral density for the honorific verbs against the control verbs extended from approximately 7 to 9 Hz for Cluster 1 and from 7 to 8 Hz and from 14 to 16 Hz for Cluster 2. We will discuss the relevance of the LIFG and the theory of mind circuit to honorification in more detail by examining the contrast in ERSP and ITC between the subject-honorific and object-honorific sentences in the three IC clusters in the following subsections.

#### 3.4.2. IC Cluster Analysis for Subject-Honorific Verbs

**Figure 12:**
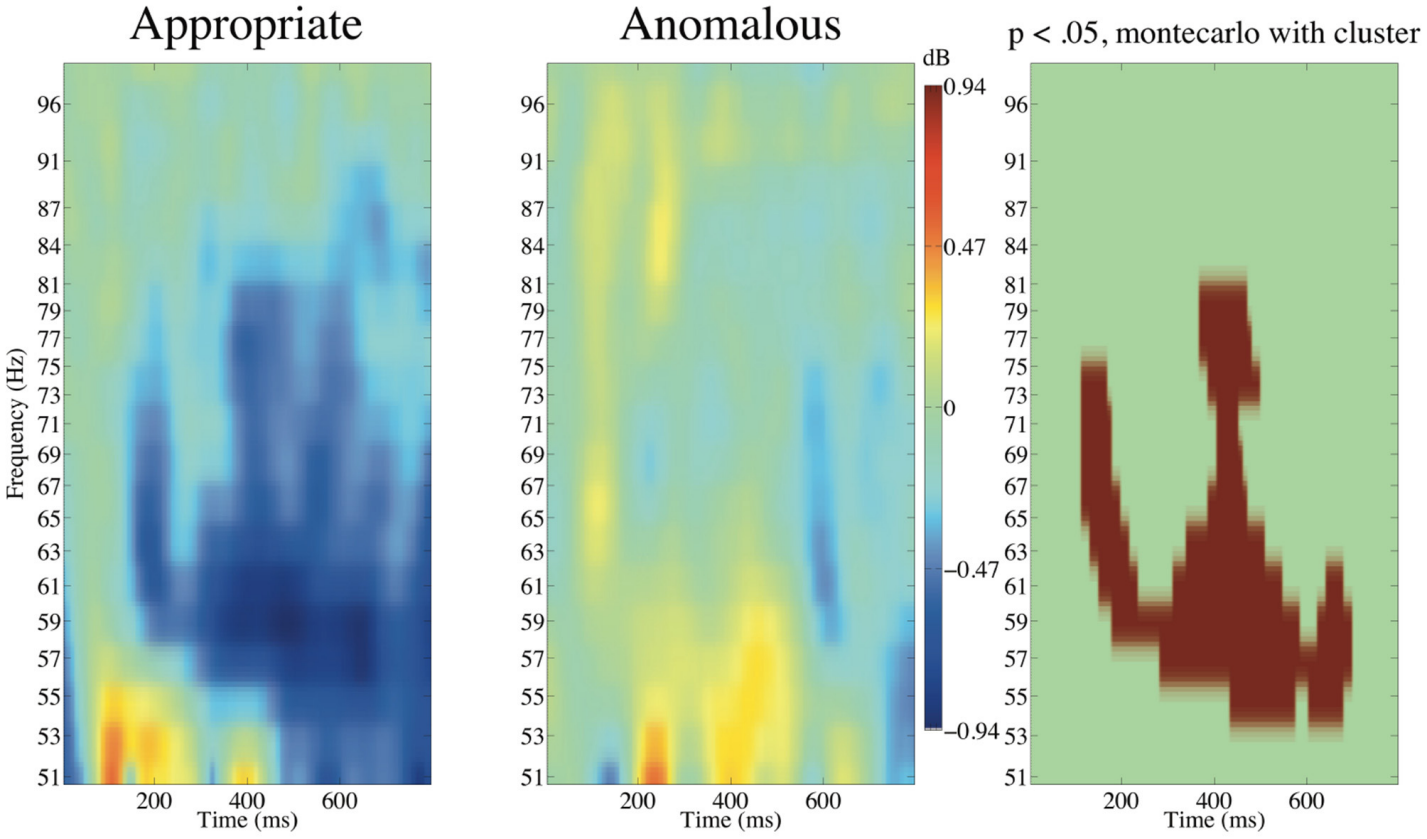
ERSP of Cluster 3 time-locked to the onset of subject-honorific verbs with the poststimulus baseline from 0 to 100 ms. The approximate extensions of the significant (*p* < 0.05) clusters in latency and frequency are indicated in red.

Figure 12 presents the ERSP of Cluster 3 time-locked to the onset of subject-honorific verbs with the baseline from 0 to 100 ms.

The cluster-based permutation test indicated that there was a significant difference between the appropriate and anomalous subject-honorific sentences in Cluster 3. A cluster of significant enhancement for the anomalous verbs against the appropriate verbs extended from approximately 100 to 700 ms for latency and from 53 to 81 Hz for frequency. We found no significant difference in ERSP for Clusters 1 or 2 and no significant difference in ITC in the three clusters.

#### 3.4.3. IC Cluster Analysis for Object-Honorific Verbs

**Figure 13:**
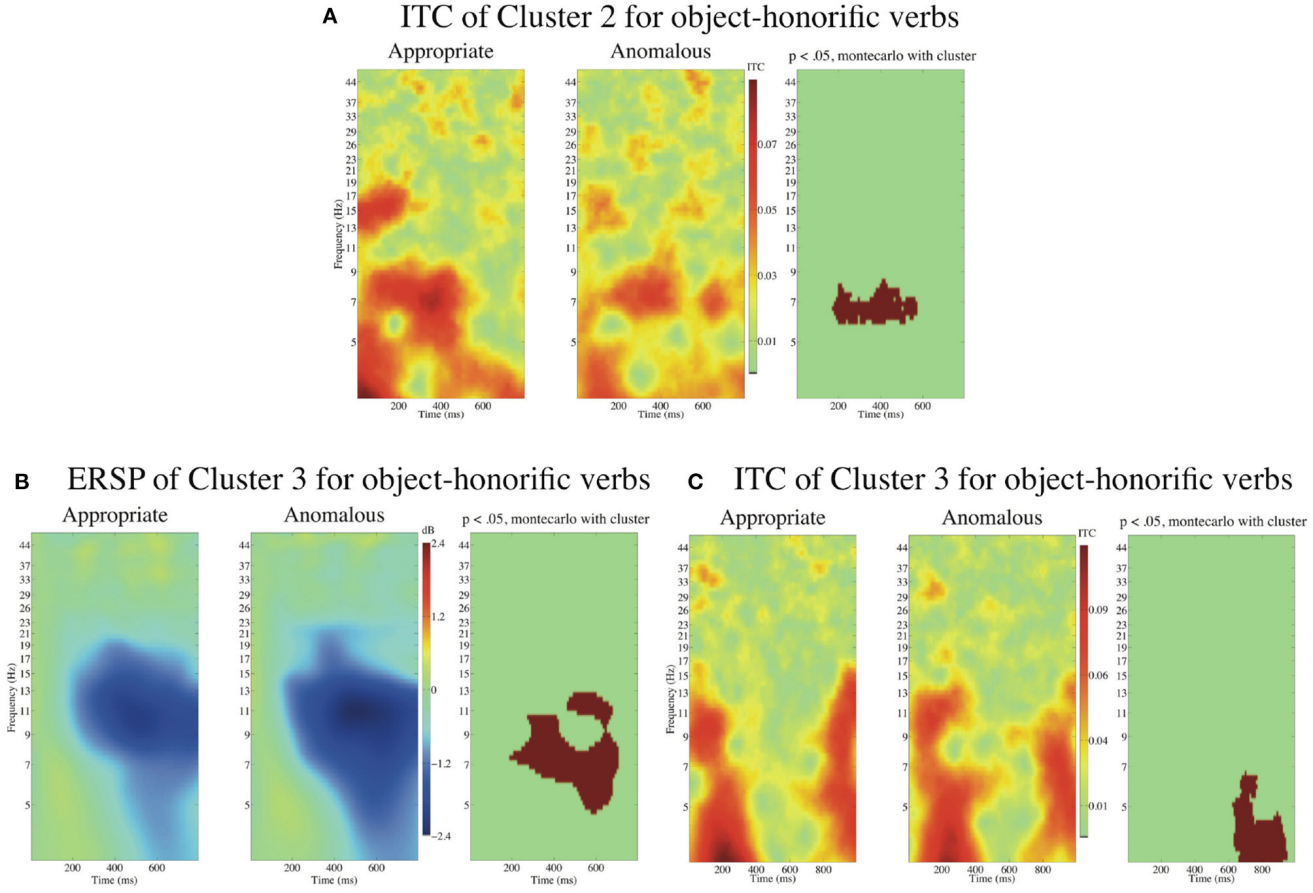
ITCs and an ERSP time-locked to the onsets of the object-honorific verbs in with the baseline from 0 to 100 ms. The ITC of Cluster 2 in **(A)**, the ERSP of Cluster 3 in **(B)**, and the ITC of Cluster 3 in **(C)** for appropriate and anomalous sentences with object-honorific verbs. The approximate extensions of the significant (*p* < 0.05) clusters in latency and frequency are indicated in red.

Figure 13 presents the ITCs and an ERSP time-locked to the onset of object-honorific verbs with the baseline from 0 to 100 ms.

The cluster-based permutation test indicated that there was a significant difference between the appropriate and the anomalous object-honorific sentences for ITC in Cluster 2 and for ERSP and ITC in Cluster 3.

A cluster of significant decrease in ITC for the anomalous sentences in Cluster 2 extended from approximately 200 to 600 ms for latency and from 6 to 8 Hz for frequency. In Cluster 3, a cluster of significant decrease in ERSP for the anomalous sentences extended from approximately 200 to 700 ms for latency and from 5 to 13 Hz for frequency, and a cluster of significant decrease in ITC for the anomalous sentences extended from approximately 600 to 900 ms for latency and from 3 to 7 Hz for frequency. We found no significant difference in ERSP for Clusters 1 or 2 and no significant difference in ITC for Cluster 1.

#### 3.4.4. IC Cluster Analysis for Anomalous Subject-Honorific and Object-Honorific Verbs

In this subsection, we present a direct comparison of the subject-honorific and object-honorific sentences. The person to be respected in a subject-honorific sentence is the subject; therefore, the sentence is judged as appropriate when we can easily find a superior-inferior relationship between the subject and the speaker. In an object-honorific sentence, on the other hand, the person to be respected is in the object; moreover, the respected person has to be superior to the person in the subject. Therefore, for an object-honorific sentence to be appropriate, a superior-inferior relationship has to be found easily between the person in the object and the speaker and between the person in the object and the subject. The human relationship coded in an object-honorific sentence can thus be more complex than that coded in a subject-honorific sentence. Therefore, we might expect a difference in the neural activity of a participant to occur when reading the two different types of honorific expressions.

**Figure 14:**
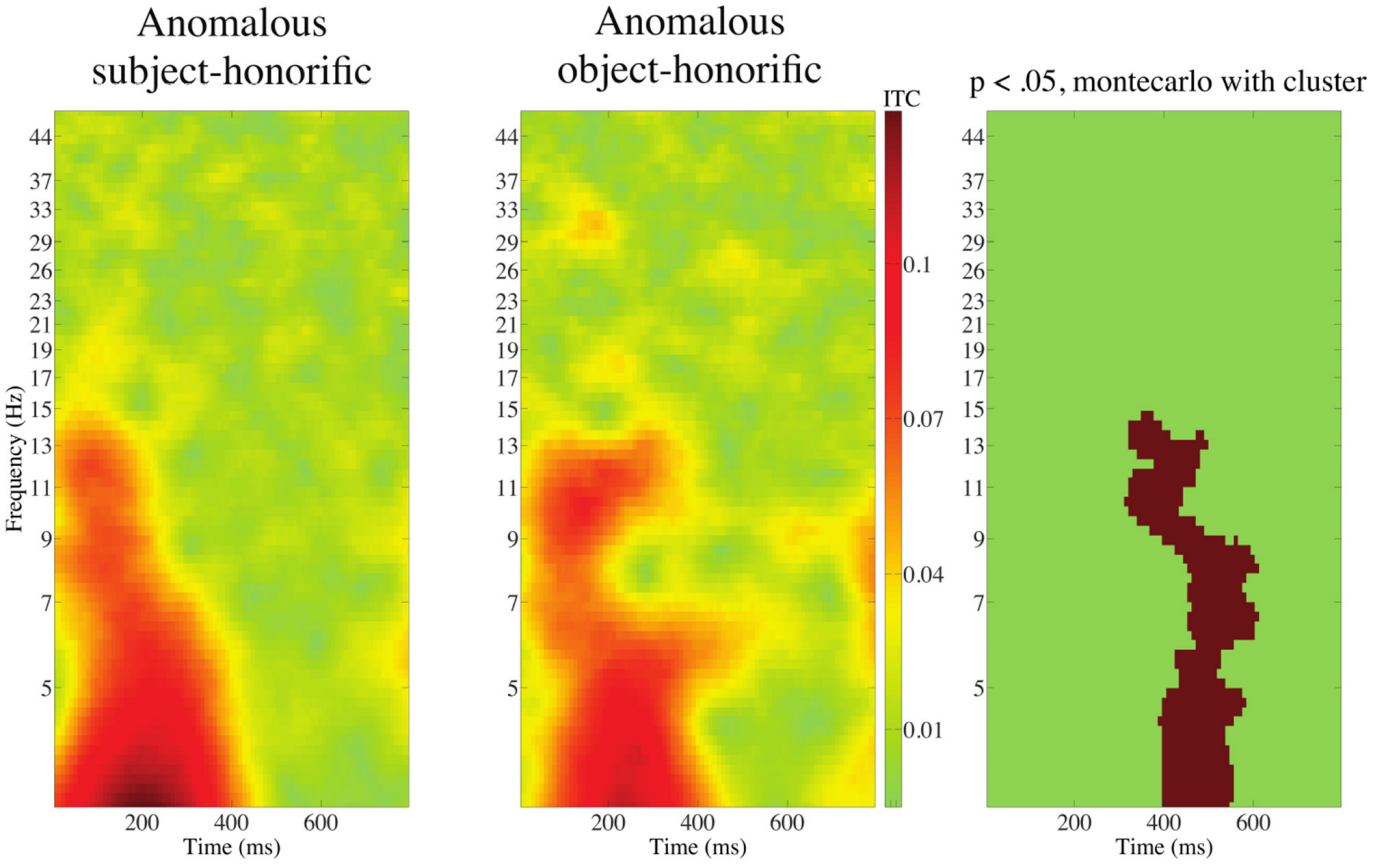
ITC of Cluster 3 time-locked to the onset of anomalous subject-honorific and anomalous object-honorific verbs. The approximate extensions of the significant (*p* < 0.05) clusters in latency and frequency are indicated in red.

Figure 14 shows the ITC of Cluster 3 time-locked to the onset of anomalous subject-honorific and object-honorific verbs.

The cluster-based permutation test indicated that there was a significant difference between the subject-honorific and the object-honorific anomalous sentences for ITC in Cluster 3. A cluster of significantly greater ITC for the anomalous object-honorific sentences in Cluster 2 extended from approximately 400 to 600 ms for latency and from 3 to 15 Hz for frequency. We found no significant difference in ERSP for the three clusters and no significant difference for Clusters 1 or 2. The greater ITC for the object-honorific verbs suggests that the processing of the object-honorific verbs, which involve more persons than the subject-honorific verbs, induces more neural activity than the processing of the subject-honorific verbs.

## 4. Questionnaire Study

As one of the anonymous reviewers correctly pointed out, the use of honorific expressions can vary among speakers depending on their personality and, more specifically, their career and domestic circumstances. To examine individual differences in the use of honorific expressions in Japanese, we conducted a questionnaire study.

We chose 128 sentences from the three hundred and sixty experimental sentences for our EEG experiment, including sixteen sentences for each of the eight honorific verbs (eight appropriate and eight anomalous sentences). We divided them into two questionnaires in a counterbalanced design with 20 filler sentences and asked participants to judge the acceptability of the sentences by choosing one of the three judgments: good, marginal, or bad.

Thirty native speakers of Japanese between 18 and 22 years old (*M* = 19.53 years, *SD* = 1.04, eleven males) participated in this study for payment. They were all undergraduate students. We assessed their sociality by using the Japanese version of the Interpersonal Reactivity Index (JIRI) (Himichi et al., 2017), which is the Japanese translation of Interpersonal Reactivity Index (IRI) by Davis (1980). The Interpersonal Reactivity Index was developed to measure the multidimensional individual differences in empathy. Four types of scales are included in (J)IRI, namely, empathic concern, fantasy, personal distress, and perspective-taking. The question items for the scale of empathic concern ask about the participants' feelings of warmth, compassion, and concern for others, and the items for the fantasy scale measure the tendency to identify with characters in movies, novels, plays and other fictional situations. The items for the personal distress scale measure the personal feelings of anxiety and discomfort that result from observing another's negative experience, whereas those for the scale of perspective-taking assess spontaneous attempts to adopt the perspectives of other people and see things from their point of view. In the JIRI, seven question items are included for each of the four dimensions, and a participant is asked to respond to each item by choosing one of five responses ranging from “describes me very well” to “does not describe me at all.” The score of each dimension is the sum of the seven scales, which ranges from seven to thirty-five. The basic statistics of JIRI for the thirty participants are presented in Table 5.

**Table 5:** Basic statistics for the 30 participants of the Japanese version of the Interpersonal Reactivity Index.

	**Mean (SD)**	**Minimum**	**Maximum**
Empathic concern	26.50 (2.79)	21	31
Fantasy	25.67 (5.38)	14	34
Personal distress	23.40 (4.88)	9	32
Perspective-taking	24.10 (4.29)	17	33

We characterized the participants by classifying them as high, middle, or low depending on their scores of each dimension. That is, a participant was considered “high” in a dimension when his/her score for the dimension was higher than the mean score by over 1/2 of the SD of the dimension (as in Table 5), whereas a participant was considered “low” when his/her score was lower than the mean score by over 1/2 of the SD. A participant whose score was between these two criteria for a dimension was considered “middle” for the dimension. Each participant thus had four classifiers corresponding to the four dimensions of the JIRI.

**Figure 15:**
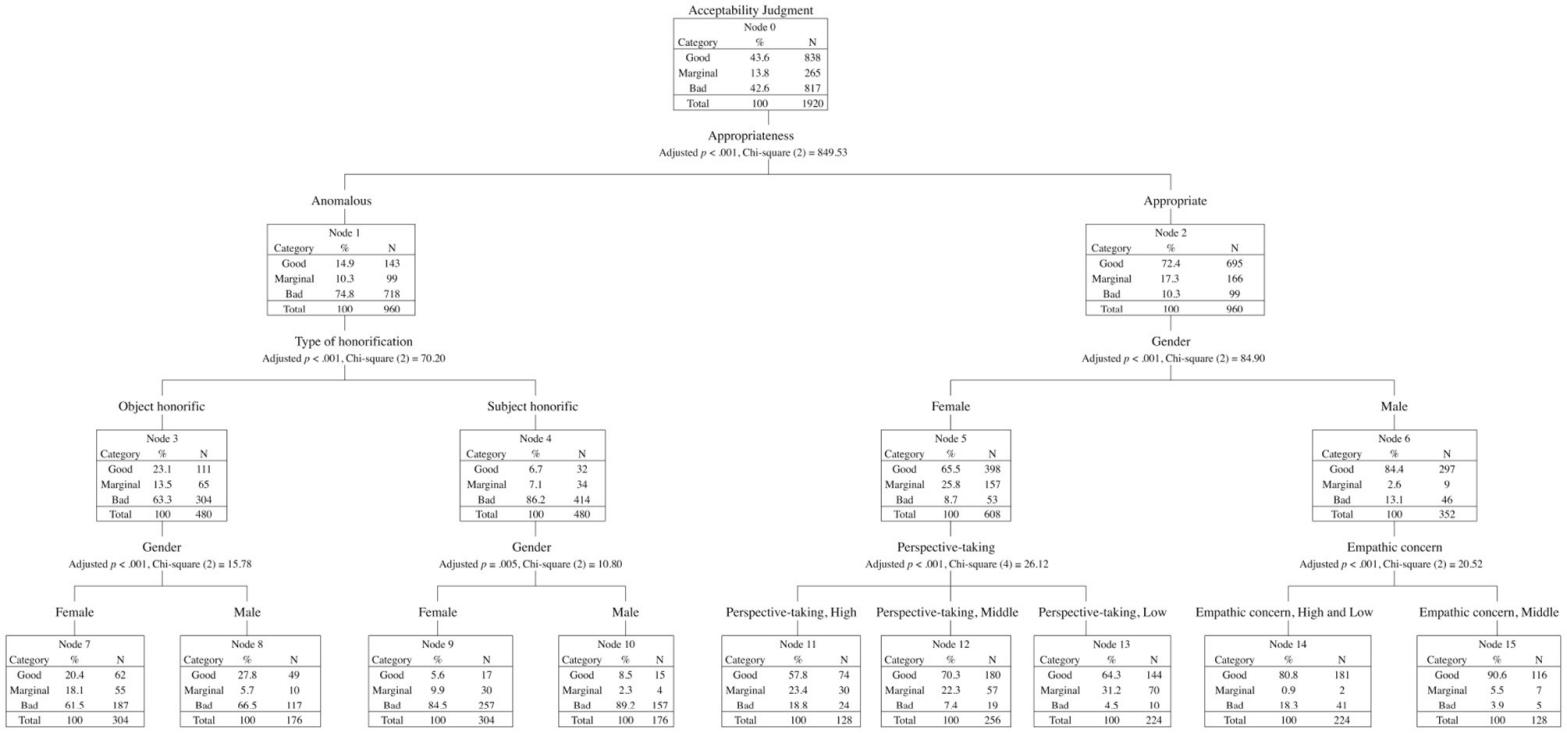
A decision tree for the acceptability judgments with the type of honorification, appropriateness, gender, and the four scales of the JIRI as independent variables.

Figure 15 shows the decision tree with the acceptability judgments (good, marginal, or bad) as the dependent variable and with the two types of honorific sentences (subject-honorific and object-honorific), appropriateness (appropriate and anomalous), gender, and the three classifiers for the four dimensions (e.g., Empathic concern, High) as independent variables. The tree was produced in SPSS version 26 (IBM).

The normatively anomalous object-honorific sentences were accepted more often than the anomalous subject-honorific sentences; this contrast was also observed in the behavioral responses of our EEG experiment (Figure 1). We find significant effects of gender for the appropriate sentences (Nodes 5 and 6) and for the (normatively) anomalous object-honorific and subject-honorific sentences (Nodes 7 and 8 and Nodes 9 and 10, respectively). We recognize that the judgments were more widely distributed for males than for females. We also find a significant effect of perspective-taking for the judgments on the appropriate sentences by females (Nodes 11, 12, and 13) and that of empathic concern for the judgments on the appropriate sentences by males (Nodes 14 and 15). It is reasonable to conclude that the gender and the sociality of a participant affected the understanding of the honorific sentences, as we assume that honorification involves social cognition.

## 5. Discussion

The anomalous sentences with subject-honorific and object-honorific verbs were incorrect according to the prescriptive grammar of Japanese. However, the participants judged the anomalous subject-honorific and anomalous object-honorific sentences to be acceptable 11 and 25% of the time, respectively, whereas they judged the ungrammatical control sentences to be acceptable only 3% of the time. In a similar fashion, the honorific sentences (normal and anomalous) in Momo et al. (2008) were judged correctly 78.4% of the time, whereas the semantic anomaly was correctly detected 93.4% of the time [the probe sentences of Jiang et al. (2013) were irrelevant to the social status information]. In our questionnaire study, again, the anomalous subject-honorific and anomalous object-honorific sentences were judged to be good 6.7 and 23.1% of the time, respectively, whereas the ungrammatical fillers were judged to be good only 2.4% of the time. The relatively high acceptable judgment rates for the anomalous subject-honorific and anomalous object-honorific sentences indicate that anomalies in the honorific expressions in Japanese are not as serious as grammatical anomalies. Furthermore, our anomalous object-honorific sentences were judged to be acceptable significantly more often than our anomalous subject-honorific sentences. Syntactic well-formedness is basically binary; that is, a sentence is well-formed when no syntactic violation is included in the sentence, whereas a sentence is fully ill-formed when it includes a syntactic violation^5^. Therefore, the relatively high acceptability of the anomalous honorific sentences in Japanese suggests that the regularity found in the honorific expressions is atypical as a syntactic phenomenon. Furthermore, the higher acceptable judgment rate for the object-honorific sentences than for the subject-honorific sentences indicates that the constraints on the Japanese honorific expressions are graded.

The first research question of this study is whether the anomalous honorific sentences in Japanese elicit an N400, as was observed in Jiang et al. (2013), or an LAN against appropriate sentences. As a result of the first analysis, Figures 3, 5 show significant negativities in parietal region for the anomalous subject-honorific verbs in the standard time window of N400 (300–500 ms) and in central region for the anomalous object-honorific verbs in the standard time windows of N400 and P600 (500–800 ms). As a result of the second analysis, Figures 4, 6 show that a cluster of significant negativity extended from approximately 290 to 350 ms in the parietal region for the anomalous subject-honorific verbs and from approximately 330 to 440 and 480 to 530 ms in the parietal region for the anomalous object-honorific verbs. We observed no significant LAN for the anomalous subject-honorific or anomalous object-honorific verbs. Molinaro et al. (2011) is suggestive here to interpret the absence of LAN in our experiment. On the basis of the review of the 29 published ERP studies on agreement violation, Molinaro et al. (2011) pointed out that an agreement relation depended on a trigger element (a subject here) and the following target element (the following verb here) and that a LAN was found when the inflectional morphology of the target constituent did not match with the value expressed in the trigger constituent. Molinaro et al. (2011) suggested that a value inflectionally expressed on the trigger was critical for eliciting a LAN and that when the value of the target was opaque, an N400-like response appeared as the index of the recruitment of higher-level representations compared to the information expressed by the functional morphology of the target word. Japanese has no other forms of verb agreement, and the morphology of honorific verb forms is not regular. Therefore, the correspondence between an honorific verb and the subject is different in nature from the agreement between a subject and the verb in person and number in English. The negativities in our experiment thus can be understood as a neural manifestation of the inconsistency between the linguistic forms and the relationship between the speaker and the addressee, as was claimed by Jiang et al. (2013), and the negativity found in both Japanese and Chinese honorification suggests that the mechanism of the integration between the linguistic forms and human relationships is, in part, cross-linguistic. Furthermore, we found a significant positivity for the anomalous subject-honorific verbs against the appropriate in the (left) centro-parietal region in the time window of 700–900 ms in the first analysis. In the second analysis, a cluster of significant positivity for the anomalous subject-honorific verbs extended from approximately 480 to 520 ms in the left frontal region and from approximately 720 to 770 ms in the parieto-occipital region. As we briefly reviewed in the section of introduction, a P600 can be elicited by a thematic violation. However, the thematic violation that elicits a P600 is the violation of animacy, as is shown in (21) again.

(21)     a.     For breakfast the eggs would only eat toast and jam (Kuperberg et al., 2003).
            b.    The hearty meal was devouring the kids (Kim and Osterhout, 2005).

The anomaly of our anomalous subject-honorific sentences is not due to the violation of animacy. The positivities in the anomalous subject-honorific sentences are thus assumed to be different from the positivity reported by Kuperberg et al. (2003) and Kim and Osterhout (2005). To the best of our knowledge, this study is the first to report parietal negativity (N400) for the honorific sentences in Japanese and a late positivity for subject-honorific sentences in the language. However, we should be careful in that these positive effects were not robust because we found no significant positive deflection in the contrast between the anomalous subject-honorific and the appropriate object-honorific verbs (Figure 7).

The second research question of this study is whether the anomalous use of the subject-honorific verb elicits a late positivity as a manifestation of the overly respectful use of the verb, as was observed in Jiang et al. (2013) for the overly respectful use of *nin/nin-de*. As Figures 3, 4 show again, we found a significant positivity for the anomalous subject-honorific verbs in the time window of 700–900 ms in the first analysis, and in the second analysis a cluster of significant positivity for the anomalous subject-honorific verbs extended from approximately 730 to 770 ms in the parietal region. These positivities for the anomalous subject-honorific verbs corresponded well with the late positivity observed in Jiang et al. (2013); therefore, we could interpret our late positivity to be a manifestation of inference in constructing a non-literal meaning from the overly respectful use of a subject-honorific verb. In contrast, this positivity was not observed for the anomalous object-honorific sentences in which the subject was disrespected relative to the person in the object, though the subject was superior to the person in the object. To the best of our knowledge, this study is the first to report late positivity that could be elicited by the inferential process to derive implicit meaning from the overly respectful use of honorific expression. Furthermore, the late positivity in this work suggests in part that the inferential processing for non-literal meaning is common to Chinese and Japanese. We should be careful again, however, in that these positive effects were not robust.

On the other hand, we did not observe late negativity for anomalous object-honorific verbs, as was reported by Jiang et al. (2013). Jiang et al. (2013) interpreted their late negativity as a manifestation of a second-pass process to reinterpret the disrespectful use of *ni* as an unintended misuse. We should note here that the disrespectful *ni* in Jiang et al. (2013) was placed in the directly quoted subordinate clause with the relevant human relationship given in the preceding main clause. By contrast, our object-honorific sentences were simple; therefore, an object-honorific verb and the corresponding subject and object were clause mates. We assume that the readers did not try to reinterpret the anomalous object-honorific verb as an unintended misuse because the relevant words were closely aligned with each other in syntax and narration, and therefore, the readers did not explore the possibility of misuse. We interpreted the absence of the late negativity for the anomalous object-honorific sentences to be due to the limited possibility of misuse in the sentence.

The third research question of this study is whether a correlation in magnitude exists between negative and positive ERPs if our anomalous sentences elicit biphasic ERPs. As Table 2 shows, the magnitudes of parietal negativity and frontal positivity for the anomalous subject-honorific verbs were positively correlated in the second analysis. As was briefly reviewed in the introduction section, several studies have demonstrated that the negative and positive ERPs elicited by semantic anomalies are negatively correlated in magnitude, whereas those elicited by syntactic processing are positively correlated. Therefore, the positive correlation between the negativity and positivity in our study suggests that the processing of Japanese honorific sentences has, in part, a syntactic property. However, no significant correlation was found for the negativity and positivity in the first analysis. We should be careful in that the correlation could be an artifact produced by the average reference in the second analysis.

The fourth research question of this study is the possible correspondence between our EEG analysis and the fMRI findings of Momo et al. (2008). Momo et al. (2008) reported significantly greater activation for honorific sentences in the LIFG, including the left lateral premotor cortex (BA6/8), angular gyrus, and precuneus, compared to that in the spelling judgment task. In our IC cluster analysis, Cluster 1 had its mean coordinate in BA6, and Cluster 3 had its mean coordinate in the medial part of the occipital region (cuneus). As Figure 11 shows, the cluster-based permutation test indicated that there was a significant difference in the ERP between the honorific and the control verbs in Cluster 1 and Cluster 3. A cluster of significant positivity for the honorific verbs against the control verbs extended from approximately 350 to 430 and 720 to 800 ms in Cluster 1 and from 350 to 500 ms in Cluster 3. Furthermore, a cluster of significantly greater power spectral density for the honorific against the control verbs extended from approximately 7 to 9 Hz in Cluster 1. As long as we accept the claim by Momo et al. (2008) that the involvement of the LIFG is a manifestation of syntactic computation, the significant positivity and the greater power spectral density in Cluster 1 suggests the presence of syntactic computation for the honorific sentences in our study. However, the significant positive deflection for the honorific verbs in Cluster 3 suggests that the circuit of the theory of mind is also involved in the processing of the honorific sentences. The normal sentences and anomalous sentences in Momo et al. (2008) corresponded to our appropriate subject-honorific sentences and anomalous object-honorific sentences, respectively; therefore, the possible differences between appropriate and anomalous sentences for both types of honorific expressions were not discussed in Momo et al. (2008).

The fifth research question of this study is whether different frequency properties exist between honorific and control sentences and between subject- and object-honorific sentences. We highlighted the possible relevance of the β band on the basis of the observation in Tokimoto and Tokimoto (2018). The cluster-based permutation test results revealed a significant difference in the frequency spectrum between the honorific and the control verbs in Cluster 2, and a cluster of significantly greater power spectral density for the honorific verbs extended from approximately 7 to 8 Hz and from 14 to 16 Hz for Cluster 2, as shown in Figure 11D. The cluster-based permutation test results revealed a significant difference between the subject-honorific and the object-honorific anomalous sentences for ITC in Cluster 3 and a cluster of significantly greater ITC for the anomalous object-honorific sentences in Cluster 2, which extended from approximately 3 to 15 Hz, as shown in Figure 14. However, the frequency properties varied between the honorific and control sentences and between the subject-honorific and object-honorific sentences; thus, we cannot be certain of the relevance of the β band in honorific processing.

Our questionnaire study demonstrated that the understanding of honorific expressions could vary depending on the speakers' gender and differences in their individual sociality, as measured by the Japanese version of the Interpersonal Reactivity Index. If we assume that the understanding of honorific expression is deeply concerned with the processing of the human relationship between the speakers, then it is natural that the acceptability judgments of honorific expressions systematically change according to the social traits of the speakers.

## 6. Limitations

We observed N400 for the anomalously used subject-honorific and object-honorific verbs, but the time window of the N400 was longer in the object-honorific than the subject-honorific verbs. Furthermore, we observed a significant positivity only for the anomalous subject-honorific verbs. The presence of the positivity for the anomalous subject-honorific verbs can be important because we observed only negativity for the ungrammatical control sentences, in which the arguments of a verb were wrongly realized. This difference in the latency of the negativity and the presence and the absence of a positivity between subject- and object-honorific sentences could be a manifestation of the different degree of acceptability of two kinds of anomalous expressions, as one of the reviewer points out. That is, the (normatively) anomalous object-honorific sentences were judged acceptable more often than the anomalous subject-honorific sentences. We cannot be decisive whether the ERP difference between the two kinds of honorific expression is qualitative or quantitative. We will leave this question for future studies.

We performed an IC cluster analysis to examine the correspondence of our experiment with Momo et al. (2008). We claimed that Japanese honorific processing was syntactic in part and that the circuit of the theory of mind was involved in processing because we found significant differences in the ERP for Cluster 1, centered in the precentral gyrus, and for Cluster 3, centered in the cuneus. However, the number of electrodes in this experiment was not large, and the dipoles were distributed widely. Another experiment with more electrodes is necessary for a more exact examination of the correspondence between this study and Momo et al. (2008).

In our IC cluster analysis, we analyzed the contrast between honorific and control verbs to examine the neural substrate for honorific processing. However, one of the reviewers suggested other possible experimental manipulations in which non-honorific verbs are compared with honorific verbs or honorifics refer to non-human objects for a better examination of the neural processing of human relationships. The possible experimental contrast would be as in (22) and (23).


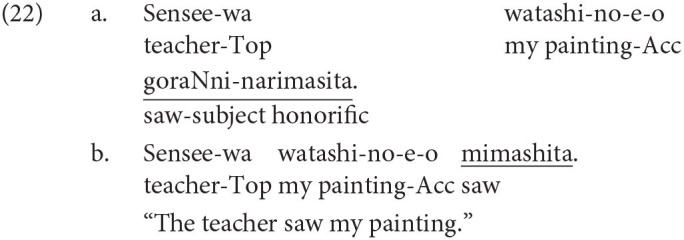



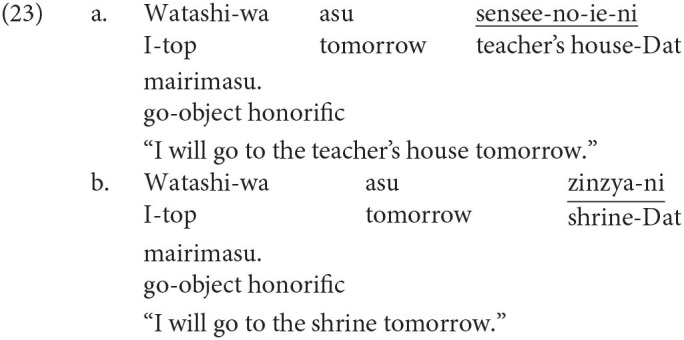


The contrast between *goraNni-narimasita* (saw-subject honorific) in (22-a) and *mimashita* (saw) in (22-b) could be used to examine honorific processing, but (22-b) can sound impolite. Therefore, we should control for differences in plausibility between the two for an exact examination of honorific processing. As for (23), the contrast at *mairimasu* (go-object honorific) can be effectively used to examine the processing of the human relationship in a sentence because *sensee* (teacher) is honored in (23-a), whereas no human is present in (23-b). However, an honorific verb presupposes the presence of a respected person, and thus, the object of respect is obscure in (23-b) (a religious entity here), though the sentence is naturally understood. Our main concern in this study is the possible contrast between subject-honorific and object-honorific sentences, paying a cross-linguistic attention to Jiang et al. (2013). We will leave further experimental manipulation for future study, which will facilitate a better understanding of the processing of the human relationship.

In the IC cluster analysis, we found a significantly greater power in the θ band for the honorific verbs than for control verbs in Cluster 1 and Cluster 2 and a significantly greater power in the β band for the former than the latter in Cluster 2. We also observed several significant differences in ERSP and ITC between the subject-honorific and the object-honorific verbs for Clusters 2 and 3. We predicted the relevance of the β band in honorific processing on the basis of Tokimoto and Tokimoto (2018) and highlighted the possible difference in ITC according to Tokimoto et al. (2019). However, no specific hypothesis for the frequency property has been given, and a theory for the exact interpretation of this property needs to be constructed. Therefore, our results should be considered descriptive.

The findings from our questionnaire study suggest that the understanding of honorific expressions are deeply connected to the speakers' gender and individual sociality. It is reasonable to assume that these social differences can be observed in neural activity. Our preliminary analysis on the perspective-taking in the Japanese sentence comprehension indicated that the individual sociality evaluated by the Autism-Spectrum Quotient in Japanese (Wakabayashi et al., 2006), the gender of participants, and their ERP amplitudes were significantly correlated variously (Tokimoto, 2019). We will leave this question for future study.

## 7. Conclusion

This study examined the neural activity related to the processing of honorific expressions in Japanese to discuss the neural substrate for the understanding of the human relationship in verbal communication as one of social cognition. Our experimental results, which correspond well with findings related to Chinese pronouns, suggest the presence of a processes to integrate the linguistic forms with the relationship between the speakers as a part of world knowledge. Furthermore, the concordance of our results with findings based on Chinese suggest that the neural process of honorification is, in part, cross-linguistic.

The processing of honorification in Japanese should have a pragmatic property because (1) negativity (N400) was observed for anomalous subject-honorific and anomalous object-honorific sentences mainly in the parietal region, (2) the LAN was not observed for these sentences, and (3) many significant differences were found in Cluster 3, centered in the medial part of the occipital region. On the other hand, several aspects of honorific processing could be grammatical because (1) we observed significant positive correlations in the magnitudes between the negativity and positivity elicited by the anomalous subject-honorific verbs (only in the second analysis), and (2) we found a significant difference in ERP for the honorific sentences against the control in Cluster 1 in the left frontal region. The integration process between the linguistic forms and human relationships should be pragmatic, but the inferior-superior relationships of the speaker and the referents should be realized in the sentences with appropriate grammatical functions. Pragmatic integration processing could thus be associated with syntactic processes, which could be why two different types of neural activities suggesting pragmatic and syntactic processes were observed.

## Data Availability Statement

The raw data supporting the conclusions of this article will be made available by the authors, without undue reservation.

## Ethics Statement

The studies involving human participants were reviewed and approved by the Ethics Committee of Mejiro University. The patients/participants provided their written informed consent to participate in this study.

## Author Contributions

ST: design of the work, acquisition of data, analysis, interpretation of data, and manuscript preparation. YM: making of the materials. NT: acquisition of data, analysis, and interpretation of data. All authors contributed to the article and approved the submitted version.

## Conflict of Interest

The authors declare that the research was conducted in the absence of any commercial or financial relationships that could be construed as a potential conflict of interest.

## Footnotes

^1^We can also refer to Lattner and Friederici (2003) and Caffarra et al. (2020) as studies that observed P600 for semantic/pragmatic anomaly.

^2^It is theoretically desirable that the person to be respected functions as a subject or an object not to be part of a complex noun phrase for the exact comparison between the subject-honorific and the object-honorific sentences. However, Japanese honorific sentences that include two persons as the subject and the object [e.g., I see (humble) the teacher, The teacher sees (exalted) me] sound unnatural and thus will require additional processing resources to retrieve the context for the plausible understanding of the sentences. One of the difficulties in making the materials was that Japanese did not have many pairs of subject-honorific and object-honorific verbs that were used frequently enough for natural understanding. We thus gave the first priority to the plausibility of a sentence in making the experimental sentences. Our experimental sentences thus included verbs of basic human behaviors, and consequently one of the two persons was part of a complex noun phrase in three fourths of the sentences.

^3^The control sentences were intended to minimize a possibility of the development of an experimental strategy by the participants. As one of the reviewers correctly points out, however, knowing that the sentences beginning with “” (I-Top) could not be an appropriate subject-honorific sentence [though half of the appropriate subject-honorific sentences began with “” (I-Gen) or “” (I-Dat)] could be a confounding factor for the examination of our research questions. As we shortly discussed in section 1, however, the subject of an appropriate object-honorific sentence can be a person mentally close to the speaker depending on the context, as in (i). (i) can be appropriate when we can assume a higher status for the manager than the father. On the other hand, “” can be the subject of an appropriate subject-honorific sentence since “my father” is superior to the speaker. We intended to control the possible fluctuation of acceptability judgments depending on the implicit context by fixing the subjects of appropriate object-honorific sentences and the non-subject persons of appropriate subject-honorific sentences as “.”

^4^In the community of EEGLAB users, around 60–70% rejection of ICs is understood to be desirable. We can thus judge that we could salvage relatively much EEG data in our preprocesses for the following analyses. As for the epoch rejection, we can refer to two of the most frequently cited ERP studies on Japanese sentence processing, namely, Ueno and Kluender (2003) and Ueno and Garnsey (2008). In Ueno and Kluender (2003), the rejection rate for eye blinks was approximately 13%, and the artifact rejection rate for other reasons was approximately 17%. In Ueno and Garnsey (2008), on the other hand, the epoch rejection rate was 6%. Our epoch rejection rates thus were relatively low, and the low rejection rates suggested the considerable effect of IC artifact rejection. ICLabel plugin we used in the preprocesses can represent the dipole location of each IC, and we present the location of one IC rejected due to a high probability of eye movements in the Supplementary Materials as an example, which is localized around the eye.

^5^Chomsky pointed out early in Chomsky (1965) that “Like acceptability, grammaticality is, no doubt, a matter of degree, but the scales of grammaticalness and acceptability do not coincide (p.11).” The distinction between grammaticality and acceptability has a long history of discussion (Sprouse and Almeida, 2013). However, the acceptability of a sentence in a pragmatic context will be more graded than its grammaticality.
