# Supplementary material for: An EEG Analysis of Honorification in Japanese: Human Hierarchical Relationships Coded in Language
Source: Front Psychol. 2021 Mar 8;12:549839. doi: 10.3389/fpsyg.2021.549839 (PMC7982684; doi:10.3389/fpsyg.2021.549839)
Supplement: Supplementary file 1 [file Data_Sheet_1.pdf]

# Supplemental Materials

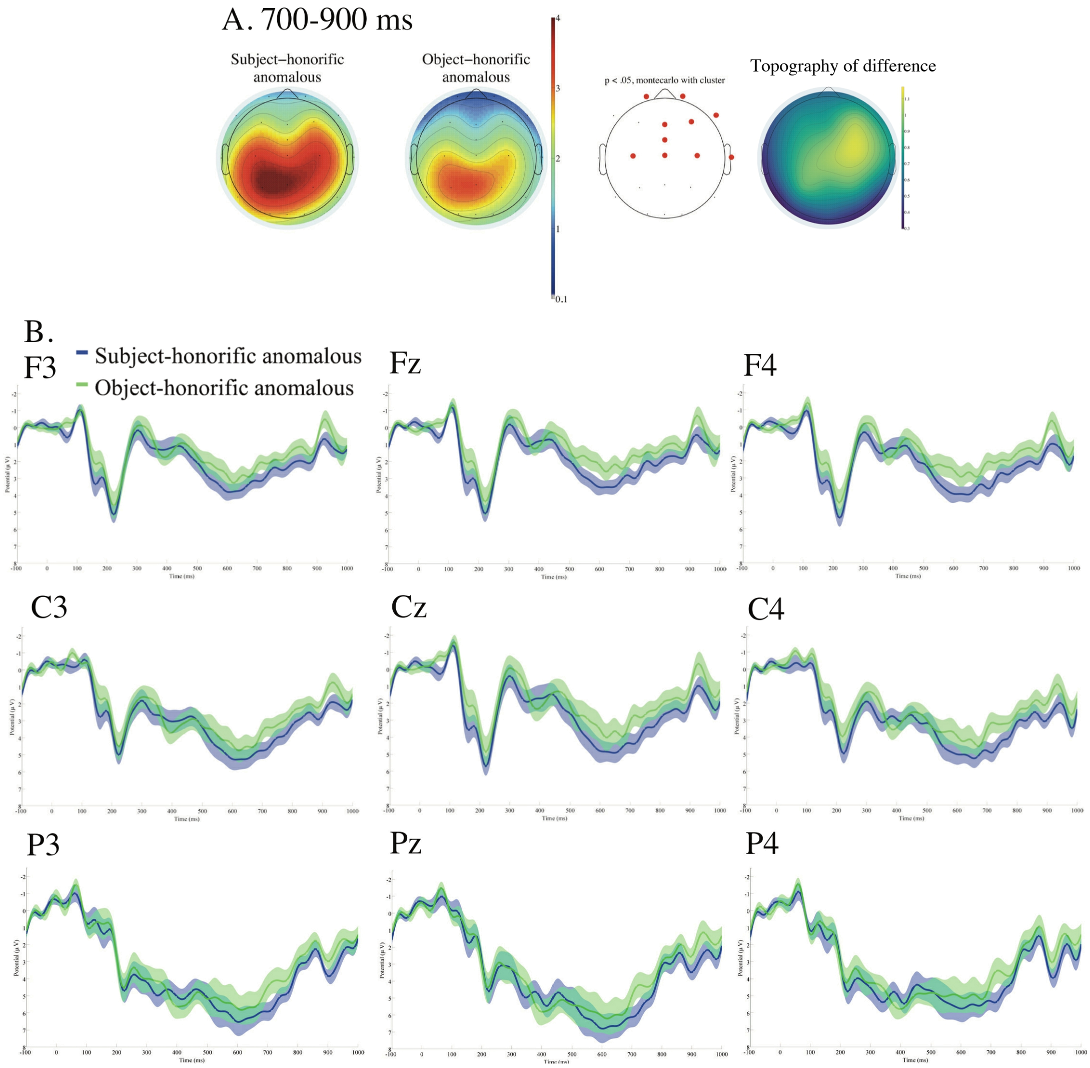


Supplemental Fig. 1: ERPs time-locked to the onsets of the verbs in the anomalous subject-honorific and the anomalous object-honorific sentences referenced to the linked earlobes **(A–B)** with the prestimulus baseline from -100 to 0 ms. **(A)** Mean topographies of the ERPs from 700 to 900 ms. The electrode sites at which significant differences were found using the cluster-based permutation test ($p < .05$) are depicted in red, and the mean topography of the difference in ERP amplitudes, for which we subtracted the amplitudes for the anomalous object-honorific condition from those for the anomalous subject-honorific condition for the time windows of 700-900 ms. **(B)** ERP waveforms at nine electrodes from -100 to 1000 ms for the two kinds of anomalous sentences, with the standard errors. Negativity is plotted upward. We find a significant positive deflection for the anomalous subject-honorific against the anomalous object-honorific sentences in the right frontocentral region in the time window of 700-900 ms.

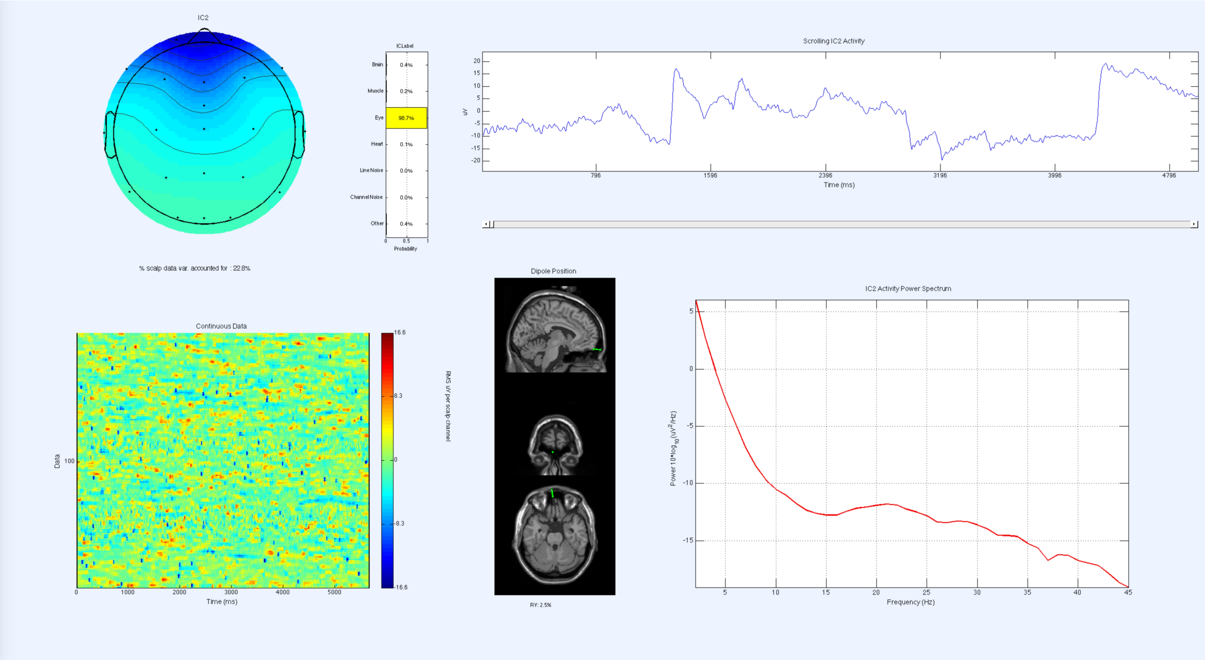


Supplemental Fig. 2: Outputs of the ICLabel plugin for one independent component rejected due to a high probability of eye movements (98.7%). We can see the dipole location localized around the eye in green.
